# Epigenetic remodeling during UV exposure: high resolution analysis of histone post-translational modifications in a DNA binding protein 2 mutant model

**DOI:** 10.1007/s00418-026-02514-5

**Published:** 2026-07-15

**Authors:** Claudio Casali, Margherita Cavallo, Adel Diaf, Davide Tunesi, Martina Furfaro, Anna Tricarico, Gloria Milanesi, Paola Perucca, Ornella Cazzalini, Marco Biggiogera

**Affiliations:** 1 Department of Biology and Biotechnology, Laboratory of Cell Biology and Neurobiology, University of Pavia, Pavia, Italy; 2 Department of Molecular Medicine, Immunology and General Pathology Unit, University of Pavia, Pavia, Italy

**Keywords:** Histone PTM, 5-methylcytosine, Perichromatin region, Chromatin organization, Ultrastructure, DDB2

## Abstract

Chromatin contains genetic information in eukaryotes. Multiple epigenetic mechanisms, such as chemical modifications of DNA and histone post-translational modifications (PTMs), regulate chromatin organization and architecture, influencing RNA transcription and driving diverse gene expression patterns from the same genome. This epigenetic control is highly dynamic and influenced by various events, such as DNA insults which cause altered gene expressions to drive aging, neurodegeneration, and cancer. However, this interplay remains poorly understood and identifying novel molecular effectors may shed light for therapeutic purposes. Here, we investigate the consequences of UV exposure in cells expressing a mutant form of damage-specific DNA-binding protein 2 (DDB2), a key protein for UV damage repair, in which interaction with PCNA is disrupted, thereby impairing proper DDB2 degradation. Our data reports that overexpression of mutant DDB2 resulted in persistent epigenetic alterations after UV irradiation, which led to elevated detection of H3K9 acetylation and trimethylation, as well as increments in 5-methylcytosine and altered subnuclear localization of γ-H2AX. Collectively, these findings demonstrate that DDB2–PCNA interaction regulates not only DDB2 stability, but also the epigenetic landscape of the genome.

**Supplementary Information:**

The online version contains supplementary material available at 10.1007/s00418-026-02514-5.

## Introduction

Most of the genetic information in eukaryotic cells is encoded within approximately 2 m of linear DNA. Steric reasons demand DNA to be packaged into a highly compact yet ordered and accessible structure. This is achieved through its association with highly conserved histone proteins, forming the complex higher-order chromatin structure. Chromatin is a dynamic and cues-responsive structure in which fundamental DNA-involving molecular processes such as transcription and replication are collectively carried out. In this context, the paradigm of epigenetic modifications is being reevaluated as both causes and consequences of DNA-related events. For instance, covalent histone posttranslational modifications (PTMs), established by specific writer enzymes, recognized by reader proteins through specialized domains, and removed by eraser enzymes, have been suggested to serve as an epigenetic code that regulates chromatin accessibility and determines the three-dimensional (3D) genome compartmentalization (Zannino et al. 2021; Wensveen et al. 2024; López-Hernández et al. 2025). Representative examples include silencing marks as trimethylated histone H3 Lys9 (H3K9me3) (Padeken et al. 2022; Zhao et al. 2023) and activating marks such as acetylated histone H3 Lys9 (H3K9ac) (Park et al. 2022; Liu et al. 2023). Furthermore, numerous histone variants exist. Their relevance is underscored by evidence linking their alteration to multiple malignancies, where they are considered to act as oncogenic drivers (Millán-Zambrano et al. 2022), as currently debated for the master DNA damage repair histone H2AX (Furuya et al. 2023; Yang et al. 2023; Contreras et al. 2024; Kelliher et al. 2024; Valceski et al. 2024).

This finely regulated environment is challenged by endogenous and exogenous sources of genotoxic stress: recent estimates suggest that eukaryotic cells may experience tens of thousands of DNA damaging events per day (Yousefzadeh et al. 2021; Fedkenheuer et al. 2025). Ultraviolet (UV) radiation is a considerable environmental hazard that threatens DNA stability in the nucleus, unfolding a cascade of events including formation of cyclobutane-pyrimidine dimers (CPDs) and pyrimidine-pyrimidone adducts (6-4PPs) that, if not properly repaired, can lead to double-strand breaks (DSBs), undermining genome integrity and leading to genetic instability, a hallmark of aging, neurodegeneration, and various pathologies including cancer (Strzałka et al. 2020; Saxena and Zou 2022; Jalan et al. 2025). Classically, epigenetics has been focused on investigation of hereditable DNA and histone modifications. Currently, evidence reports that certain forms of DNA damage are able to induce persistent changes in the transcriptional states of cells, influencing gene expression and signaling pathways. However, a deep comprehension is still missing, since secondary effects have been reported whereby alterations in the epigenome promote changes in expression patterns of RNA processing factors, influencing abundancy and availability of key nuclear components such as heterogeneous nuclear ribonucleoproteins (hnRNPs) (Burgess et al. 2012; Min et al. 2022; Paull and Opresko 2026). These fine-tuned mechanisms are modulated by several factors. While parameters such as cell cycle stage and senescence have been extensively studied (Gorbunova et al. 2007; Hustedt and Durocher 2017), increasing attention is being directed towards the role of local nucleoplasmic organization, chromatin context, and underlying epigenetic modification under UV damage. (Jeon et al. 2024; Polyzos et al. 2024; Vergara et al. 2024; Ambrosio et al. 2025; Kaya and Adebali 2025; Mathis et al. 2025). Important questions remain unresolved, such as how UV exposure reshapes cells epigenome and which proteins may contribute to this process. For this purpose, we took advantage of an in vitro model of HEK293 cells expressing a mutant form of damage-specific DNA-binding protein 2 (DDB2), a protein known for its central role in recognizing UV-induced lesions, unable to bind PCNA, an essential effector in driving DDB2 degradation (Cazzalini et al. 2014). We observed that these cells, after UV-damage, show a delay in DNA repair, increased survival, cell growth, motility, and invasion abilities (Perucca et al. 2018, 2024). Here, we report profound nucleoplasmic alterations, including changes in DNA methylation and histone PTMs.

## Material and methods

### Cell lines and transfection

HEK293 (human embryonic kidney) cell line was purchased from the ECACC (code 85120602) (CLS Cat# 300,192/p777_HEK293, RRID: CVCL_0045). The cell line was cultured in Dulbecco’s modified Eagle’s medium (DMEM, Sigma-Aldrich, St. Louis, MO, USA) supplemented with 10% fetal bovine serum (Life Technologies-Gibco), 2 mM L-glutamine (Life Technologies-Gibco), 100 U/ml penicillin, 100 μg/ml streptomycin, in a 5% CO_2_ atmosphere.

Cells were stably transfected with DDB2^Wt^ or its mutant form DDB2^PCNA−^; this mutant form is produced by site-specific mutagenesis in PIP-box sequence and so it is not able to interact with PCNA, as previously reported (Cazzalini et al. 2014). The DDB2 expression level in HEK293 cells was assessed in our previous published paper (Perucca et al. 2015) and confirmed in the present work (Supplementary Data). Parental (referred to as control, CTR) and stably transfected HEK293 clones were exposed to UV-C irradiation (10 J/m^2^, 20 s) using a TUV lamp (Philips) at a fluence rate of 0.5 J/m^2^/s at 254 nm, as measured by a DRC-100X radiometer (Spectronics) in all the experiments, in order to induce DNA damage and activate a DDB2-dependent DNA damage response. All the experiments were carried out at least three times.

### Sample preparation for EM

For transmission electron microscopy (TEM) experiments, cells were collected 3 and 7 days after UV irradiation performed under the same protocols reported above. Only for γ-H2AX analysis, cells were collected 1 and 72 h after damage induction. Cells were detached by trypsinization, centrifuged at 150 *g* for 10 min, and then fixed with 4% paraformaldehyde at room temperature (RT) for 30 min and then at 4 °C for 1 h and 30 min. After fixation, cells were rinsed with PBS, preembedded in 2% agarose, and incubated in 0.5 M NH_4_Cl at 4 °C for 30 min. The samples were dehydrated using graded ethanol, embedded in acrylic resin (LR White, Agar Scientific, Stansted, UK) and finally sectioned on a Reichert OM-U3 ultramicrotome. Ultrathin Sects. (60–80 nm) were collected on 200 mesh nickel grids for immunogold labeling and on gold grids for osmium ammine DNA staining. The specimens were observed with a JEM 1200 EX II electron microscope (JEOL, Peabody, MA, USA) operating at 100 kV and equipped with a MegaView G2 CCD camera (Olympus OSIS, Tokyo, Japan).

### EM immunogold labeling

For the immunolabeling, ultrathin sections were incubated in normal goat serum (NGS) (Sigma-Aldrich, St. Louis, MO, USA) diluted 1:100 for 3 min, and subsequently in a drop of primary antibody (Table 1). Grids were rinsed in PBS/TWEEN20 and in PBS, incubated in NGS, and then in a drop of the corresponding gold particle-conjugated secondary antibody (Jackson ImmunoResearch, West Grove, PA, USA). After that, the specimens were rinsed in PBS and in water. As negative controls, the same protocols were followed using equal volumes without the primary antibody (Casali et al. 2026).

**Table 1:** List of antibodies used for EM immunogold labeling. Corresponding utilization details and references are indicated

Target	Host	Dilution	Incubation	Ref.
5mC	Mouse	1:500	Overnight at 4 °C	GTX629448—AB_2888114 (GeneTex)
H3K9ac	Rabbit	1:20	Overnight at 4 °C	GTX88007—AB_10731164 (GeneTex)
H3K9me3	Rabbit	1:20	Overnight at 4 °C	GTX121677—AB_10721938 (GeneTex)
p300	Mouse	1:10	2 h at RT	SC-48343—AB_628075 (Santa Cruz Biotechnology)
Phospho-histone H2AX (Ser 139)	Mouse	1:20	2 h at RT	05–636—AB_309864 (Sigma-Aldrich)
6 nm Colloidal Gold AffiniPure^TM^ Goat Anti-Mouse IgG	Goat	1:20	30 min at RT	115–195–166—AB_2338729 (Jackson ImmunoResearch)
12 nm Colloidal Gold AffiniPure^TM^ Goat Anti-Mouse IgG	Goat	1:20	30 min at RT	115–205–068—AB_2338730 (Jackson ImmunoResearch)
12 nm Colloidal Gold AffiniPure^TM^ Goat Anti-Rabbit IgG	Goat	1:20	30 min at RT	111–205–144—AB_2338016 (Jackson ImmunoResearch)

### EDTA regressive staining

Ultrathin sections were stained using the ethylenediaminetetraacetic acid (EDTA) regressive technique. Briefly, grids were incubated in 4% uranyl acetate for 2 min and then rinsed in water. After that, specimens were incubated in 0.2 M EDTA for 30 s, rinsed in water, incubated in lead citrate for 2 min, and finally thoroughly rinsed in water.

### Osmium ammine staining

Ultrathin sections were stained using the osmium ammine technique for DNA-specific (Biggiogera et al. 2024) labeling, rendering DNA electrondense for quantitative analysis. Grids were incubated in 5 N HCl at RT for 30 min to generate free aldehyde groups. After rinsing, the grids were incubated in a solution of osmium ammine-B (Polysciences, Warrington, PA, USA) for 1 h and then thoroughly rinsed.

### Flow cytometry

Control and stably transfected HEK293 clones overexpressing DDB2^Wt^ or DDB2^PCNA−^ were plated (1 × 10^6^) in 90-mm culture dishes, irradiated, and fixed with methanol after 1 and 72 h. Subsequently, cells were washed with PBS and blocked in PBST buffer (PBS, 0.2% Tween 20) containing 1% bovine serum albumin (BSA). The samples were incubated for 1 h with the specific mouse antibody anti-phospho histone H2AX (Ser 139) 1:100 (Millipore Cat# 05–636, RRID: AB_309864), diluted in PBST buffer/BSA. After washing, each reaction was followed by incubation for 30 min with anti-mouse conjugated with Alexa Fluor 488 Dye (Molecular Probes). The cells were analyzed for each sample with a Partec PAS II flow cytometer.

For cell cycle analysis, nonirradiated and irradiated HEK293 DDB2^Wt^ and DDB2^PCNA−^ cells were collected 72 h after UV-induced DNA damage and fixed in 70% ethanol. After rehydration, DNA staining was obtained using the FxCycle^TM^ Violet Stain kit (ThermoFisher Scientific, Waltham, MA, USA) at RT for 30 min. An Attune NxT Acoustic Focusing flow cytometer (ThermoFisher Scientific, Waltham, MA, USA) was used for the analysis of stained samples.

### Western blot analysis

Control and stably transfected HEK293 clones overexpressing DDB2^Wt^ or DDB2^PCNA−^ protein were exposed to UV-C irradiation. After 3 and 7 days from UV-induced DNA damage, pelleted cells were collected. For blot analysis, the cells were directly lysed in SDS sample buffer (65 mM Tris–HCl pH 7.5, 1% SDS, 30 mM dithiothreitol (DTT), 10% glycerol, 0.02% Bromophenol Blue).

Proteins were resolved by sodium dodecyl sulfate polyacrylamide gel electrophoresis (SDS-PAGE) (4–20% precast gel, Bio-Rad) and membranes probed with anti-Actin 1:1000 (Sigma-Aldrich Cat# A4700, RRID: AB_476730), anti-H3K9me3 1:1000 (Santa Cruz Biotechnology Cat# sc-130356, RRID: AB_2118015), and anti p-48DDB2 (Rockland Cat# 100–401-A10, RRID: AB_2276988) primary antibody; and anti-mouse HRP 1:10,000 (Sigma-Aldrich Cat# A9044, RRID: AB_258431) and anti-rabbit HRP 1:10,000 (Sigma-Aldrich Cat# A9169, RRID: AB_258434) secondary antibody. The signal was revealed using enhanced chemiluminescence by Azure c600 Gel Imaging System (Azure Biosystems).

### Cell growth after UV-irradiation

Control and stably transfected HEK293 clones overexpressing DDB2^Wt^ or DDB2^PCNA−^ were seeded (3.5 × 10^4^) in six-well plates after UV-C irradiation. Cell growth rate was determined by counting the number of cells as a function of the time by using a hemocytometer (Heinz Herenz Hamburg).

### Statistical analysis and data processing

For western blot analysis, the quantification of the bands was performed compared with the loading control (Actin) on the same blot using ImageJ (Rueden et al. 2017) (RRID: SCR_003070). For EM immunolabeling, at least 30 nuclei per condition were analyzed. Three replicates were performed for each marker and colloidal gold grains presence and distribution were evaluated by two independent operators. For the osmium ammine analysis, electron micrographs were processed by applying a Gaussian blur filter (radius of 1), and the extent of heterochromatin relative to the total nuclear area was calculated by thresholding using the triangle algorithm. For statistical analysis, either one-way analysis of variance (ANOVA) with Tukey’s post-hoc test or Mann–Whitney *U* test was performed using GraphPad Prism version 5.03 (GraphPad Software, La Jolla, CA, USA). Results are reported as mean values with standard error of the mean (SEM), with *P* values from *P* < 0.05 considered statistically significant (*P < 0.05; **P < 0.01; ***P < 0.001). Unless differently stated, data are shown normalized to the respective controls for coherent visualization. Images were processed using Fiji (RRID: SCR_002285). Where explicitly stated, colloidal gold grains were postprocessed with false coloring for readability. Contrast and brightness of TEM micrographs were adjusted according to the standard of ethics in image processing. Tables were assembled using Jasc Paint Shop Pro version 7.02.

## Results

### p300 and H3K9 acetylation increase in DDB2^PCNA−^ cells after UV exposure

**Fig. 1:**
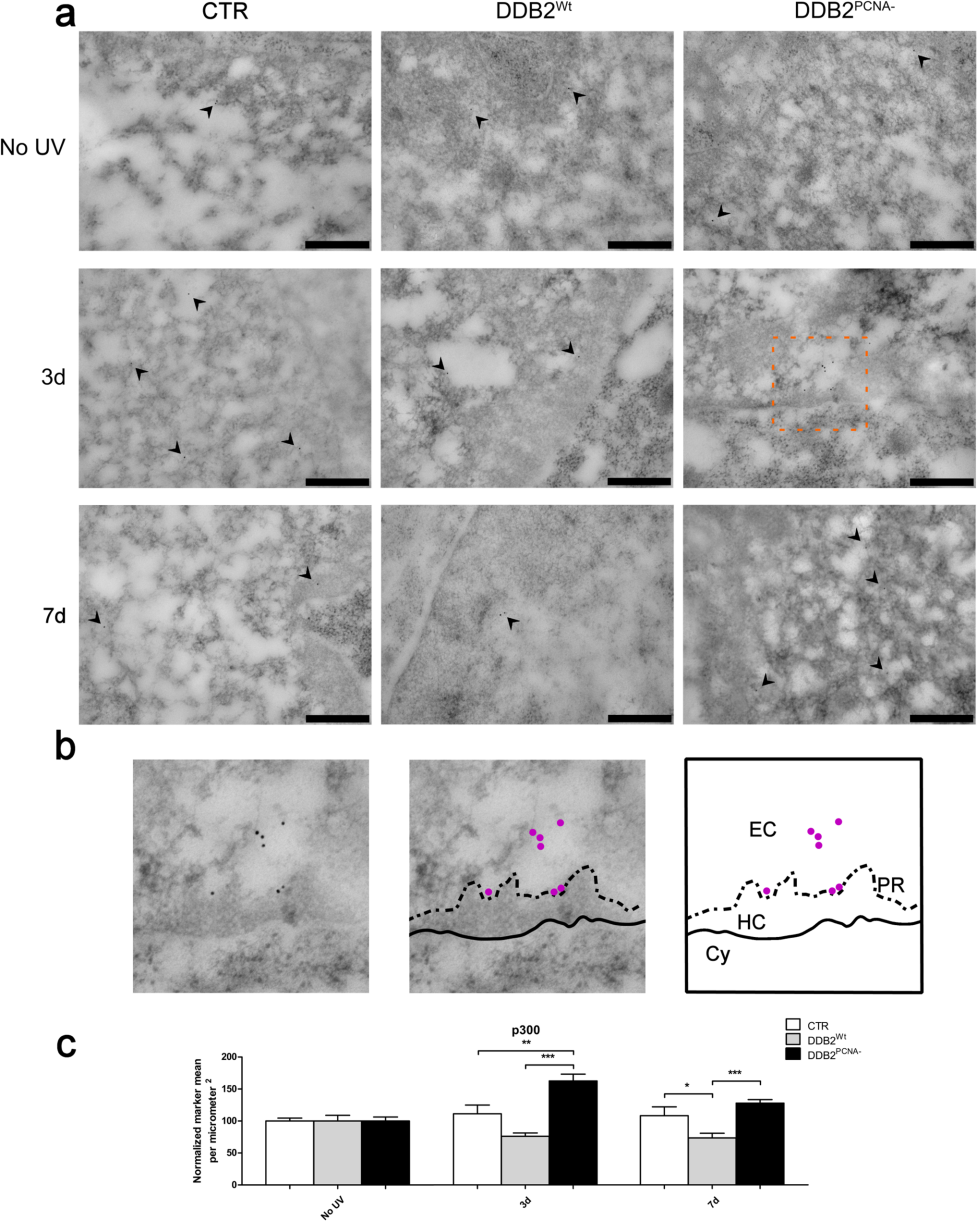
Acetyltransferase p300 following UV stress. **a** Representative EM micrographs of EDTA-stained HEK293 cells labeled for p300 (arrowheads). Scale bars: 500 nm. **b** High-magnification inset (postprocessed and schematized) representing EM immunolabeling and the appearance of the EDTA-stained nucleus. Contrarily to conventional EM micrographs, heterochromatin (HC) appears decontrasted. p300 labeling (purple dots) is mainly localized to euchromatin (EC) and the perichromatin region (PR). Cy, cytoplasm. Dash-dot line indicates the boundary between HC and PR. **c** Normalized marker mean of p300 per micrometer square in nuclei in absence of UV damage and at 3 and 7 days postirradiation. *P* values: not significant *P* > 0.05; *P < 0.05; **P < 0.01; ***P < 0.001. Exact *P* values are available in Supplementary Material

The acetyltransferase p300 modulates genome stability by regulating chromatin organization, promoting the expression of target genes involved in cell growth and homeostasis maintenance, and facilitating the accessibility of DNA repair proteins (Kikuchi et al. 2023; Wu et al. 2025). Based upon its role in shaping the epigenetic landscape, we evaluated p300 specific nuclear abundance by EM immunogold labeling (Fig. 1a and Fig. 1b). Our analysis revealed a significant increase in nuclear p300 labeling in cells expressing DDB2^PCNA−^ at 3 days postirradiation. This result is consistent with the elevated proliferation rate observed in this cell line. On the contrary, cells expressing either endogenous DDB2 or overexpressing DDB2^Wt^ did not exhibit statistically relevant differences at this time point. Interestingly, DDB2^PCNA−^ expressing cells maintained a similarly elevated labeling for p300 at 7 days post-UV, suggesting a potential persistent form of epigenetic alteration that may be absent when DDB2 is functional (Fig. 1c).

**Fig. 2:**
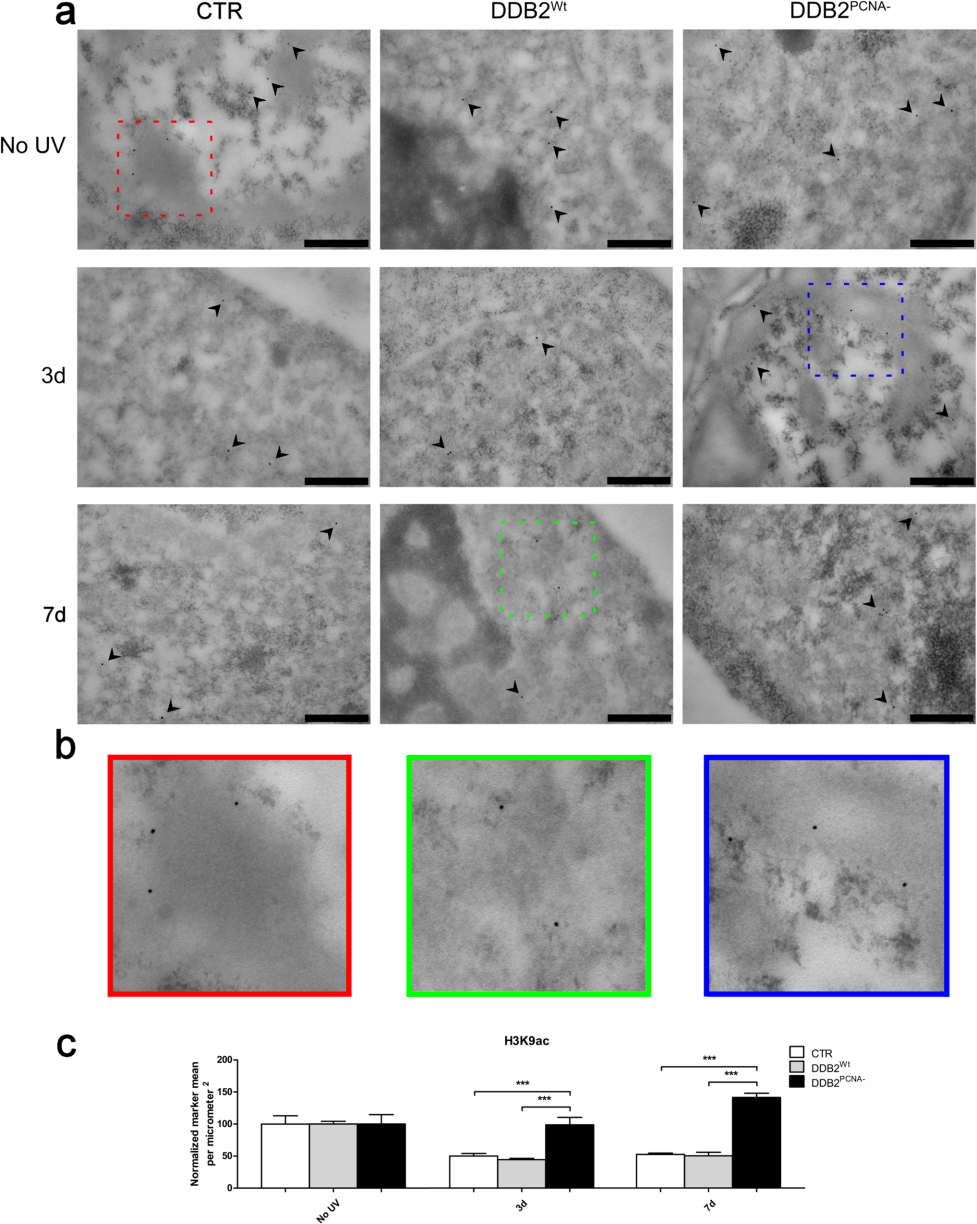
H3K9 acetylation exhibits concordance with p300 distribution. **a** Representative EM micrographs of EDTA-stained nuclei following immunogold labeling for acetylated H3K9. Scale bars: 500 nm. **b** Insets displaying the consistent ultrastructural localization of H3K9ac at the euchromatin–heterochromatin interface across cell types and time points, consistent with its role in promoting chromatin decondensation and transcriptional accessibility. **c** Normalized marker mean of H3K9ac per micrometer square in the nucleus highlights higher labeling in DDB2^PCNA−^ cells compared with the other conditions. *P* values: not significant *P* > 0.05; ***P < 0.001. Exact *P* values are available in Supplementary Material

Next, to further investigate this epigenetic response, we examined the levels of acetylated histone H3 at lysine K9 (H3K9ac), a marker associated with chromatin relaxation and enhanced DNA accessibility (Fig. 2a). Immunogold labeling revealed that H3K9ac immunolabeling is primarily localized to euchromatin and the perichromatin region (Fig. 2b), consistent with its role in promoting chromatin decondensation for active gene transcription. Mirroring the pattern observed for p300, DDB2^PCNA−^ expressing cells exhibit the highest labeling of H3K9ac at both 3 and 7 days postirradiation, indicative of a sustained epigenetic trend in this cell line. Once more, no statistically relevant differences were observed in cells overexpressing functional DDB2, whether at basal or higher levels (Fig. 2c). These data suggest that, at least with respect to this form of histone modification, the distinct epigenetic impact resides on the integrity of DDB2–PCNA interaction, rather than on the absolute expression level of DDB2. This supports the hypothesis of a more profound regulatory role for DDB2–PCNA binding that extends beyond structural scopes.

### Diverse temporal response of histone methylation in DDB2^PCNA−^ and DDB2^Wt^ cells

**Fig. 3:**
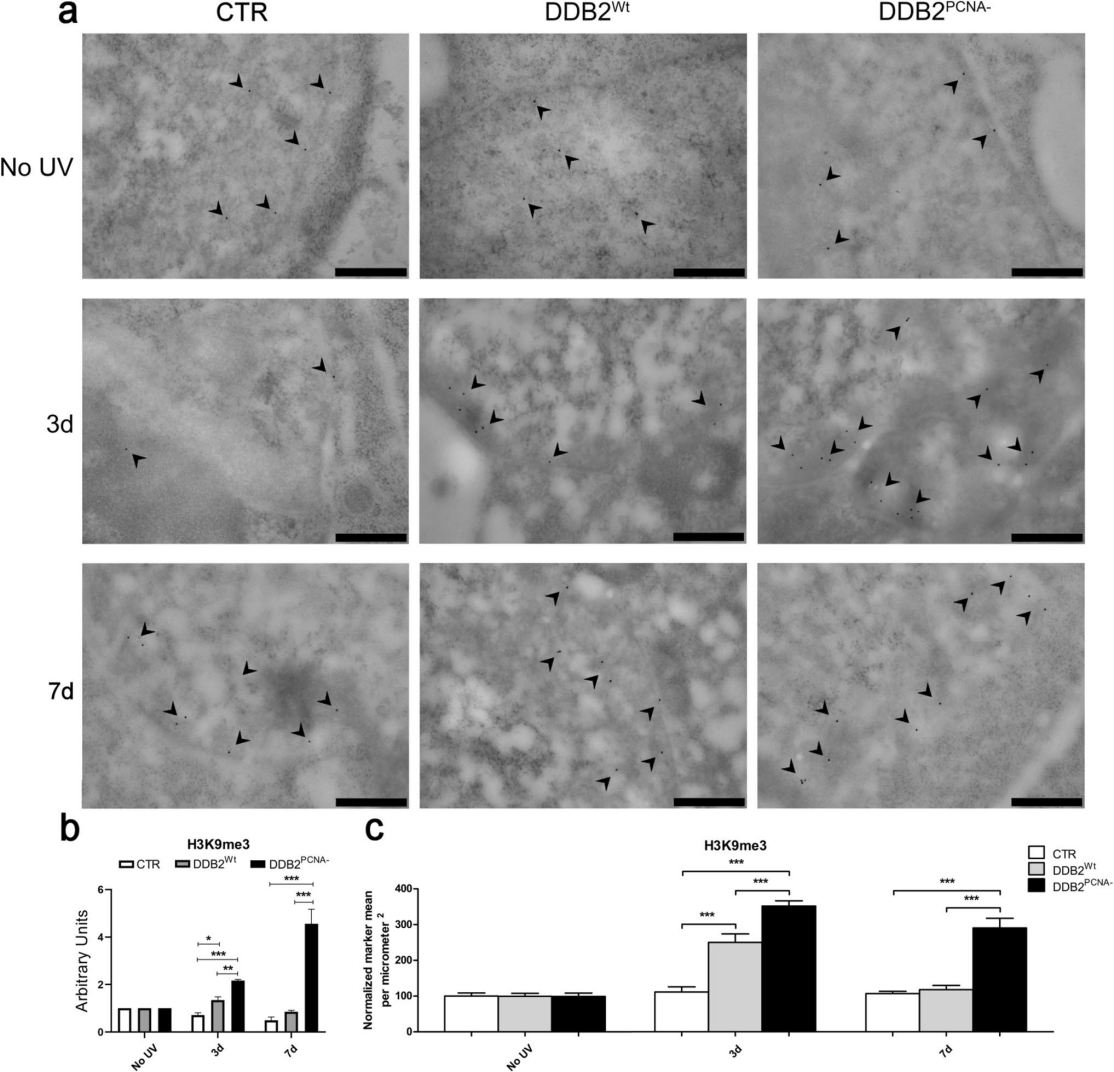
Time-dependent modulation of H3K9 trimethylation following UV-induced damage. **a** Representative micrographs of EDTA-stained nuclei following immunogold labeling for H3K9me3. Note the preferential heterochromatin localization. Scale bars: 500 nm. **b** Quantifications of western blot. **c** marker mean per micrometer square in the nucleus report a stable increase in H3K9me3 levels in DDB2^PCNA−^ from day 3 onward. In contrast, DDB2^Wt^ cells are characterized by a transient rise on day 3 that reverts to control levels by day 7. *P* values: not significant *P* > 0.05; *P < 0.05; **P < 0.01; ***P < 0.001. Exact *P* values are available in Supplementary Material

Since we reported relevant alterations in processes related to histone acetylation, we extended our analysis to histone methylation to gain a more comprehensive understanding about the epigenetic state of the cell. For such reasons, we focused our attention on histone H3 lysine 9 trimethylation (H3K9me3), a histone PTM implicated in the regulation of many biological processes, including the formation and maintenance of transcriptionally silent heterochromatin (Fig. 3a). First, we performed western blot analysis which revealed increased levels of H3K9me3 following UV-induced damage, particularly in DDB2^PCNA−^ expressing cells. A significant increase was already present at 3 days postirradiation, with the highest level detected at 7 days, approximately four-fold with respect to control cells. In contrast, cells overexpressing DDB2^Wt^ also showed a significant increase at 3 days post-UV, but these levels ​​returned to baseline by day 7 (Fig. 3b and Supplementary Data). This temporal profile suggests that the expression of the mutant DDB2 leads to persistent heterochromatin clumps formation, maintained over time of these experiments. To explore the spatial aspects of this methylation mark, we performed EM immunolabeling of H3K9me3. Subnuclear localization appeared specific within condensed chromatin, supporting its role in heterochromatin formation. Quantification of gold particles was in strong agreement with western blot data and further revealed a time-dependent course specific to DDB2^Wt^ overexpressing cells, characterized by a transient increase in H3K9me3 at 3 days postirradiation, which declined to control levels at day 7 (Fig. 3c). This unexpected time-dependent abundancy suggests a different form of epigenetic response to the UV damage subordinate to the effective DDB2–PCNA binding, where their interaction is critical for fine-tune regulating H3K9 trimethylation over time. Disruption of this interaction results in prolonged methylation, suggesting a potential failure in the epigenetic landscape reset.

### DNA and histone methylation display a similar time-dependent course

**Fig. 4:**
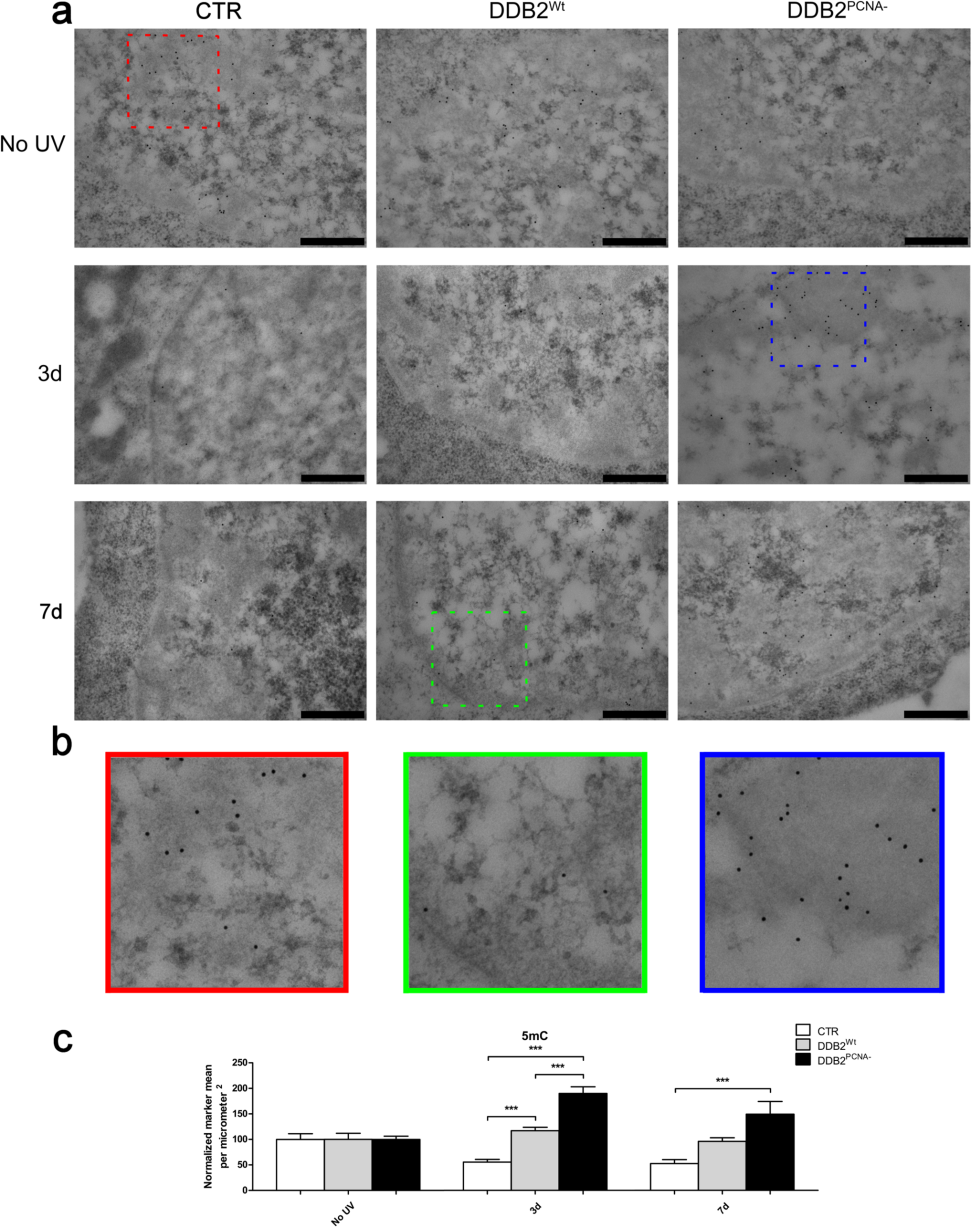
5-methylcytosine exhibits a notable parallelism with trimethylated H3K9. **a** Representative micrographs of EDTA-stained nuclei following 5mC immunogold labeling. Similarly to H3K9me3, DNA methylation is preferentially localized to heterochromatin regions. Scale bars: 500 nm. **b** Insets displaying the consistent ultrastructural localization of 5mC in condensed chromatin domains. **c** Normalized marker mean per micrometer square in the nucleus. Both DDB2^Wt^ and DDB2^PCNA−^ showed increased labeling at 3 days post-UV compared with control conditions. However, by day 7, 5mC levels in DDB2^Wt^ cells revert to the prestress control conditions, while the increment is stably maintained in DDB2^PCNA−^ cells. *P* values: not significant *P* > 0.05; ***P < 0.001. Exact *P* values are available in Supplementary Material

To assess whether the temporal dynamics observed in histone methylation were mirrored in changes at the DNA level, we investigated the presence of 5-methylcytosine, a key epigenetic mark commonly linked to chromatin compaction (Fig. 4a). TEM immunolabeling revealed a well-defined parallelism with the profile of H3K9me3, both in terms of subnuclear localization and colloidal gold grains count. Across all time points, at the nanoscale level, 5mC appeared to be generally associated with the heterochromatin regions (Fig. 4b), consistent with its most diffused interpretation as a marker of chromatin condensation. Quantifications showed that DDB2^PCNA−^ expressing cells exhibited an increase in 5mC at 3 days post-UV, which was maintained through to day 7. In contrast, cells overexpressing DDB2^Wt^ shared an analogous significant increase on day 3, which however was transient, reverting to the control level by day 7 (Fig. 4c).

These findings suggest a tight correlation between histone and DNA methylation marks in response to UV-induced stress. The coordinated and reversible pattern in DDB2^Wt^ overexpressing cells suggests the presence of a regulated epigenetic program that relies on the DDB2–PCNA interaction.

### DNA damage, chromatin organization, and phosphorylated histone H2AX

**Fig. 5:**
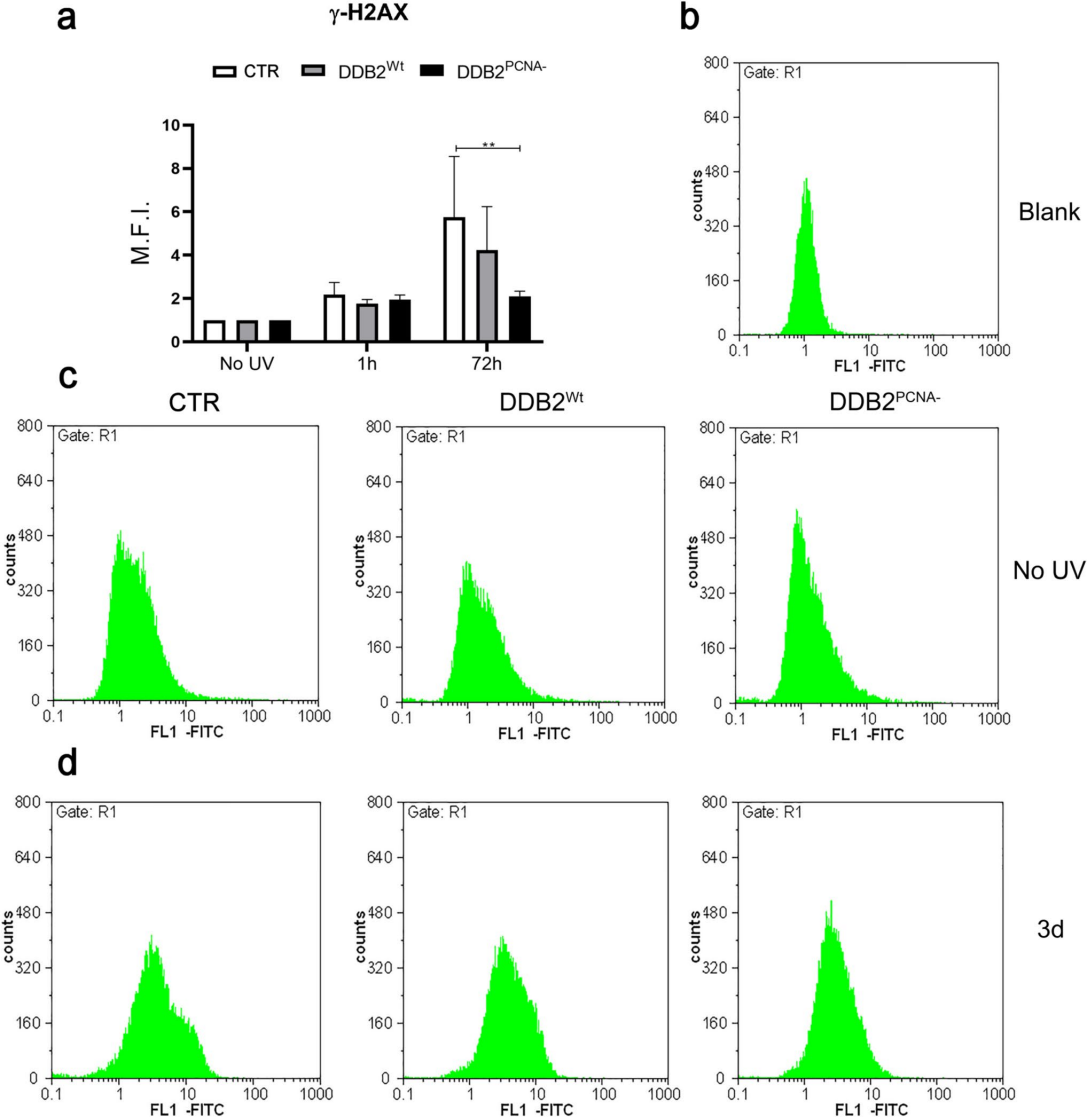
UV damage activates H2AX. **a** Mean fluorescence intensity (MFI) quantification of γ-H2AX analyzed via flow cytometry at 72 h postirradiation. A significant increase in γ-H2AX is observed in control and DDB2^Wt^ expressing cells, while DDB2^PCNA−^ cells show no significant increase compared with nonirradiated conditions. *P* values: not significant *P* > 0.05; **P < 0.01. Exact *P* values are available in Supplementary Material. **b–d** Representative flow cytometry histograms of the three cell lines without UV irradiation and 72 h post damage. x-axis: γ-H2AX fluorescence at 488 nm; y-axis: cell count

**Fig. 6:**
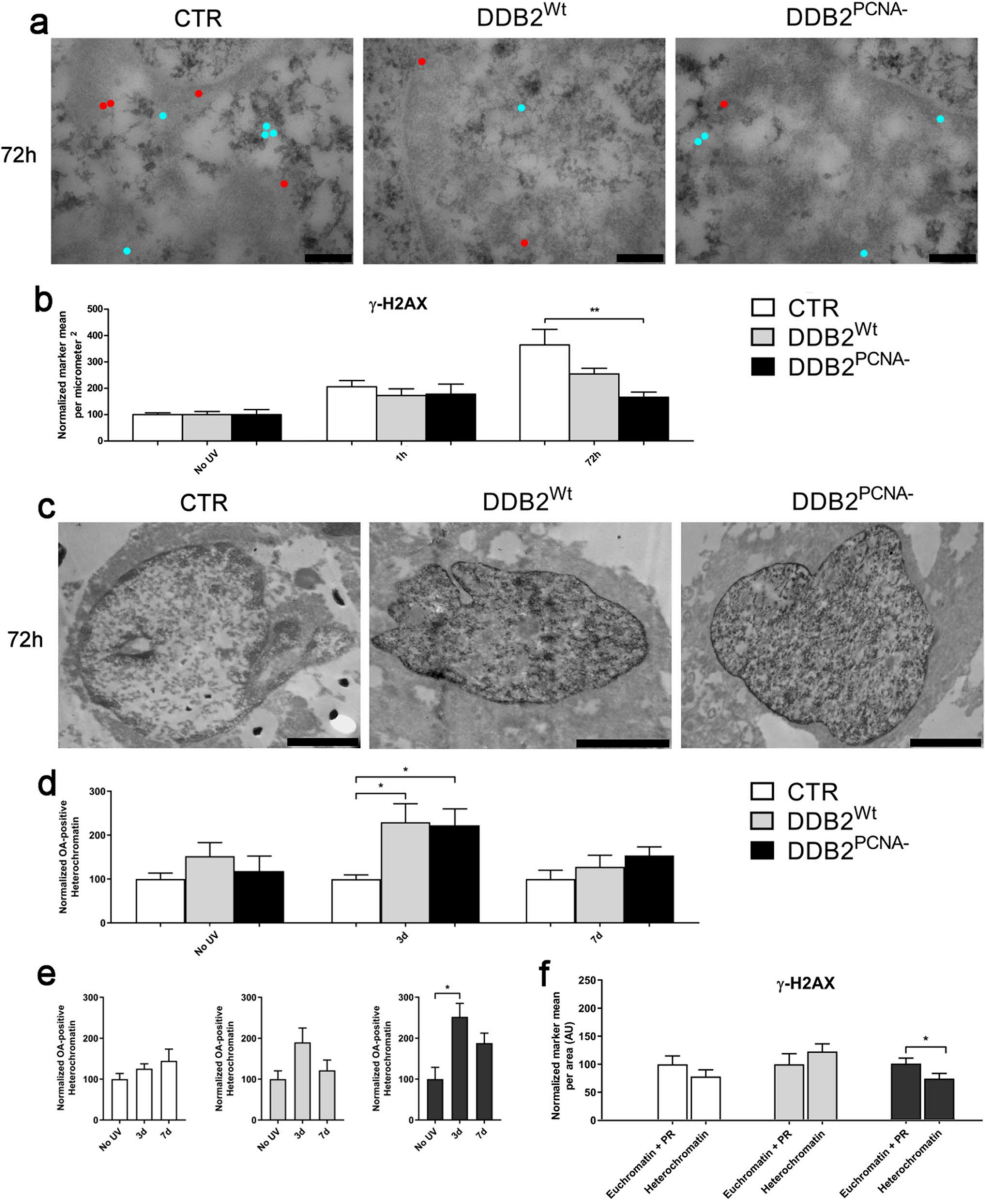
Subnuclear localization of γ-H2AX. **a** Representative EM micrographs of EDTA-stained nuclei at 72 h postirradiation highlighting γ-H2AX immunogold labeling. In DDB2^PCNA−^ expressing cells, labeling is primarily retrieved in sterically accessible nuclear compartments such as euchromatin and the perichromatin region (cyan labeling) compared with the low grain count in heterochromatin (red labeling). In contrast, γ-H2AX signal in control and DDB2^Wt^ expressing cells is sparse and diffuse. Scale bars: 200 nm. **b** Quantification of γ-H2AX labeling in the whole nucleus. **c** Representative EM micrographs of osmium ammine-stained DNA at 72 h postirradiation, with increased electron density in both cell lines overexpressing DDB2. Scale bars: 2 µm. **d** Quantification of chromatin compaction across the three considered time points. Data normalized to CTR to emphasize differences at the level of the various cell lines. **e** Normalized quantification of condensed chromatin per cell nucleus in control cells (white histograms), DDB2^Wt^ and DDB2^PCNA−^ overexpressing cells (grey and black histograms, respectively). **f** Subnuclear distribution analysis between decondensed (Euchromatin + PR) and condensed (Heterochromatin) areas at 72 h, confirming statistically significant enrichment of activated H2AX in the decondensed chromatin domains in cells expressing DDB2^PCNA−^. *P* values: not significant *P* > 0.05; *P < 0.05; **P < 0.01. Exact *P* values are available in Supplementary Material

In a wide range of eukaryotic organisms, the phosphorylated form of the histone variant H2AX (γ-H2AX) is a canonical marker for DNA damage, and it is involved in assembly of DNA repair proteins at damaged chromatin regions (Prabhu et al. 2024). We therefore performed a flow cytometric analysis at different recovery times after UV irradiation to study the γ-H2AX activation as a signal target for DNA damage (Fig. 5a and Fig. 5b). No significant alterations were observed between nonirradiated samples (Fig. 5c) and those collected 1 h after irradiation (data not shown). However, at 72 h after damage, relevant differences emerged (Fig. 5d). At this time point, cells expressing DDB2^PCNA−^ displayed the lowest γ-H2AX levels, significantly reduced compared with irradiated control cells, while no statistically relevant difference was identified in respect to nonirradiated samples. Notably, UV-irradiated control cells and DDB2^Wt^ overexpressing cells reported a significant increase in the activation of histone H2AX at 72 h compared with nonirradiated samples. Moreover, these observations were further confirmed by immunogold labeling analysis, which revealed an analogous scenario (Fig. 6a and Fig. 6b). Collectively, these data support the idea that cells expressing mutant DDB2 protein display an alteration in the DNA damage signaling, as reflected in lower γ-H2AX levels that are commonly associated with a reduction in the efficiency of DNA damage response activation.

Although a defined relationship exists between the activation of H2AX and the efficiency in damage repair, its subnuclear localization remains poorly characterized at the ultrastructural level. Hence, given the previously investigated role of DDB2 in the epigenetic context, in particular when assessing histone PTMs, we hypothesized that its presence and functional state might influence the spatial pattern of H2AX activation. First, we performed an analysis of the nucleoplasm by osmium ammine staining aimed at evaluating potential differences in DNA condensation. Our data indicated that chromatin organization reflected the UV-altered epigenome. In particular, at 72 h post UV-damage, cells overexpressing DDB2^Wt^ and DDB2^PCNA−^ were characterized by higher extension of osmium ammine-positive regions (Fig. 6c, Fig. 6d, and Fig. 6e). This is consistent with increased detection of H3K9me3 in both cell lines, altogether with diminished H3K9ac and increase in 5mC signals in DDB2^Wt^ and DDB2^PCNA−^ cells, respectively. Second, immunogold labeling revealed that cells expressing DDB2^PCNA−^ displayed a unique γ-H2AX pattern (Fig. 6f). γ-H2AX localized with statistical significance in decondensed and accessible nuclear compartments, such as euchromatin and perichromatin region, despite the increased chromatin condensation. This distribution was statistically divergent from that observed in control and DDB2^Wt^ overexpressing cells, which in contrast, exhibited sparser and more diffuse labeling. This data is particularly relevant taking in consideration that, at the analyzed time point, the euchromatin-to-heterochromatin ratio is comparable in the cell lines overexpressing DDB2, limiting the likelihood of stochastic distributions in the underlying chromatin organization and strengthening the concept of specific patterns. The distinctive labeling of γ-H2AX in DDB2^PCNA−^ expressing cells is on par with a defective resolution of the DDR, as most sterically accessible nuclear domains are marked but not efficiently cleared of repair signals, suggesting persistent signaling without effective resolution.

### General effects of UV exposure in control and mutant cells

**Fig. 7:**
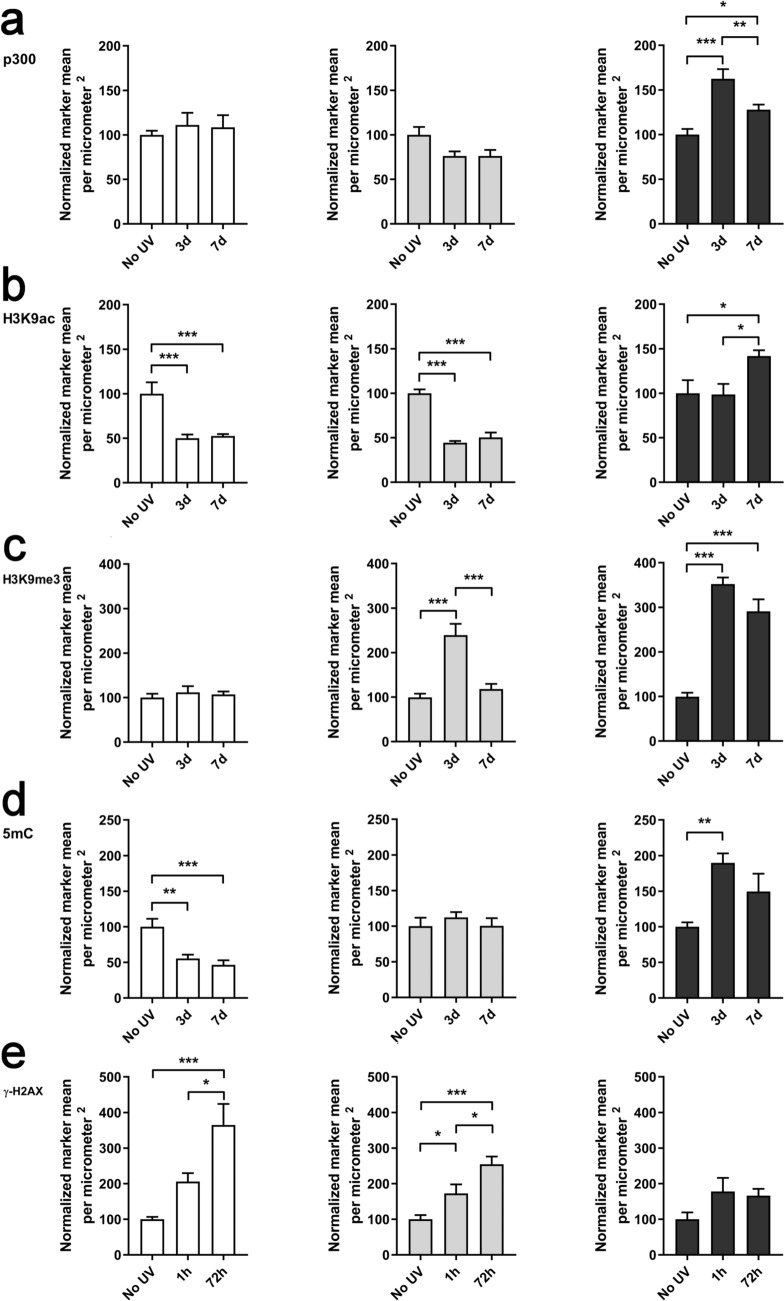
**a** Normalized marker mean of p300 per micrometer square in nuclei in control cells (white histograms), DDB2^Wt^ and DDB2^PCNA−^ overexpressing cells (grey and black histograms, respectively). **b** Normalized marker mean of H3K9ac per micrometer square in control cells, DDB2^Wt^ and DDB2^PCNA−^ overexpressing cells. **c** Normalized marker mean of H3K9me3 per micrometer square in control cells, DDB2^Wt^ and DDB2^PCNA−^ overexpressing cells. **d** Normalized marker mean of 5mC per micrometer square in control cells, DDB2^Wt^ and DDB2^PCNA−^ overexpressing cells. **e** Normalized marker mean of γ-H2AX per micrometer square in control cells, DDB2^Wt^ and DDB2^PCNA−^ overexpressing cells. *P* values: not significant *P* > 0.05; *P < 0.05; **P < 0.01; ***P < 0.001. Exact *P* values are available in Supplementary Material

Finally, we evaluated the overall variations of epigenetic marks focusing our attention on the level of the single cell lines, to give a general point of view over the effect of the UV exposure on the investigated model (Fig. 7). For this reason, we plotted the presented data following a time-wise comparison in cells expressing basal level of DDB2, overexpressing DDB2^Wt^ and DDB2^PCNA−^. Our data highlighted an evident diverse response to the UV damage with, in particular, differences on the level of DDB2^PCNA−^ overexpressing cells. Considering the acetyltransferase p300 (Fig. 7a), no significant alterations were reported following UV exposure neither in control nor in DDB2^Wt^ overexpressing cells, while we reported a significant increase in the nuclear labeling in the DDB2^PCNA−^ cell line following UV irradiation. These findings are in strong correlation with H3K9ac (Fig. 7b) course. In fact, H3K9ac labeling was reduced following UV damage in all conditions but cells overexpressing DDB2^PCNA−^, where not only is there no reduction, but the signal increased, coherently with the higher maintained levels of p300. Considering the repressive histone PTM H3K9me3 (Fig. 7c), compared with controls, the data highlighted an increased labeling in cells overexpressing DDB2^Wt^ and DDB2^PCNA−^ at the first analyzed time point after UV damage. However, analyzing the second point, while the former cell line reestablished levels comparable to the predamage ones, the latter maintained higher signal. This data is consistent with the hypothesis of the loss of a fine-tune regulation of H3K9 trimethylation over time following UV exposure and failure reset the epigenetic landscape in the nucleoplasm. This aspect is partially confirmed by DNA methylation analyses assessing 5mC (Fig. 7d) that showed two opposite trends, with a reduction in control cells and an increase in cells overexpressing DDB2^PCNA−^. Finally, assessing γ-H2AX (Fig. 7e) our data confirmed the contrast between cell lines. While cells expressing basal levels of DDB2 or overexpressing the wild-type form showed a constant increase after UV damage, the scenario is different in cells overexpressing DDB2^PCNA−^ that maintained basal labeling of γ-H2AX. This data further supported that presence mutated DDB2 correlated with altered DNA damage signaling and reduced efficiency in activation of DNA repair.

## Discussion

Epigenetic modifications are linked with regulation of gene expression patterns (Gershman et al. 2022; Davidovich et al. 2026). However, their causal contribution and spatially dependent impact remain to be elucidated. In particular, adaptation to cellular stresses is reported to coordinate transcriptional and post-transcriptional reprogramming involving various alterations in the epigenome, including histone PTMs. Understanding how the epigenome responds to stressful signals, such as UV damage, and which proteins contribute to this process may shed light on potential novel therapeutic targets and approaches (Watt et al. 2025; Plessier et al. 2026). To investigate this question, we utilized an in vitro model based on HEK293 cells that were chosen for the intrinsically low basal level of DDB2, thereby facilitating experimental modulation of both its expression and function. DDB2 plays a pivotal role in the recruitment of DNA repair factors via interaction with PCNA, which is in turn essential for DDB2 degradation. Cells were stably transfected with constructs encoding either wild-type DDB2 (DDB2^Wt^) or a mutant variant unable to bind PCNA (DDB2^PCNA−^), with expression levels previously validated (Perucca et al. 2015). Following stable integration, cells were exposed to UV-C irradiation. All cell lines remained viable and proliferative over the first week postirradiation. The cellular growth was analyzed by two different experimental approaches (Supplementary data), reporting that cell proliferation is conserved across all time points considered; moreover, flow cytometry analysis confirmed that the S-phase is similar in all samples. In particular, DDB2^PCNA−^ expressing cells exhibited the highest proliferation rate among the experimental groups, consistent with previous observation (Perucca et al. 2018).

Data obtained using a DDB2 mutant model highlighted that the epigenome is reshaped following UV irradiation. This supports the idea that the interaction between DDB2 and PCNA is not limited to structural and stability purposes, rather it has a profound role in such reorganization. Specifically, disruption of DDB2 binding to PCNA was associated with an increased H3K9ac, coherent with an increased p300 nuclear abundance, together with elevated 5-methylcytosine levels and H3K9me3, the latter even 1 week after UV damage. In contrast, cells overexpressing functional DDB2 showed a response primarily involving histone methylation, characterized by a transient and reversible increase in H3K9me3. This pattern is consistent with a more controlled and adaptive chromatin response to the insult. Overall, overexpression of mutated DDB2 suggested the failure to reset the epigenome to a preirradiation condition. Chromatin organization is tightly interconnected to its epigenetic state (Casali et al. 2022b; Zannino et al. 2023; Policarpi et al. 2024; Altıntaş et al. 2024; Lin et al. 2025), as clearly visualized by the specific (Biggiogera et al. 2024) osmium ammine technique. High levels of histone acetylation, including acetylated H3K9, are correlated with reduced local chromatin compaction and increased steric accessibility (Halsall et al. 2021; Casali et al. 2024). This structural state favors DNA accessibility to transcription and repair factors: highly decondensed regions are thus associated with elevated local transcriptional activity, and DNA lesions are more efficiently recognized and repaired (Lorat et al. 2012; Ortega et al. 2021; Aricthota et al. 2022; Downs and Gasser 2024; Zencir et al. 2025). Oppositely, H3K9 trimethylation and 5-methylcytosine have been described as playing central roles in chromatin condensation and transcriptional repression (Mattei et al. 2022; Casali et al. 2022a; Kreibich and Krebs 2023; Zhou et al. 2025). The concomitant increase in both permissive and repressive marks observed in DDB2^PCNA−^ cells therefore suggests a substantial reorganization of chromatin states rather than a simple unidirectional shift toward activation or silencing.

This is particularly relevant in light of our findings on γ-H2AX. First, we reported that preventing DDB2 binding to PCNA led to lower levels of γ-H2AX. Low levels of γ-H2AX have been found to be correlated with epithelial–mesenchymal transition (EMT) (Liu et al. 2022), a key process in tumor invasiveness and metastasis formation by granting proliferative advantage (Perucca et al. 2024), through repression of key epithelial genes such as *CDH1*, *RAB25*, *SERPINB5*, and *MAGI1*, as well as activation of mesenchymal genes including *SNAI2* (Slug), *ZEB1, VIM, THBS1, VCAN, TGFB2, ITGB4* (Weyemi et al. 2016a, b). Activation of *Slug* and *ZEB1* has also been linked to increased detection of positive histone PTMs (Lindner et al. 2020; Zhang et al. 2022; Fernandez-De-Los-Reyes et al. 2023), as also confirmed in this study with H3K9ac. This strongly supports the potential role of γ-H2AX in regulating EMT, EMT-related transcription factors, and chromatin organization through epigenetic modifications. Then, in contrast to most published studies, we focused on γ-H2AX subnuclear localization at ultrastructural resolution. Our data indicate that stable interaction between DDB2 and PCNA is essential to guarantee proper γ-H2AX localization and distribution in the nucleus. Normally, γ-H2AX is removed once the lesions are resolved (Valdiglesias et al. 2013; Dardare et al. 2024; Dibitetto et al. 2024). Specifically, after UV damage, sterically accessible regions are enriched in γ-H2AX foci that are shortly after disbanded (Rawal et al. 2022; Wang et al. 2024). Interestingly, preventing PCNA–DDB2 interaction resulted in γ-H2AX preferential detection within regions characterized by high steric accessibility, such as euchromatin and the perichromatin region (Fakan and van Driel 2007; Cavallo et al. 2026). This redistribution may indicate altered efficiency of the DNA damage response (Cowell et al. 2007; Oizumi et al. 2024): it is tempting to speculate that this may be potentially driven by altered chromatin signaling dynamics affecting recognition and resolution of DNA lesion (Silva and Ideker 2019; Steurer et al. 2022; Donnio and Giglia-Mari 2025).

We acknowledge several limitations of the current study. First, our findings are specific to the in vitro model utilized and should not be directly extrapolated to other physiological contexts. Second, because data were obtained from a single cell line carrying the mutation under investigation, they should not be generalized to other cell types beyond HEK293 cells. In this model, DDB2 binding to PCNA is disrupted, and we provided evidence that this affects damage recognition and repair, nucleoplasm organization, and the epigenome. Future studies could determine, for instance, whether disrupting other protein–protein interactions within analogous pathways results in comparable epigenetic effects. Third, although high-resolution microscopy provides a valuable tool for spatial information and subnuclear investigations, it does not define underlying genetic mechanisms. Coupling this approach with complementary functional assays would contribute to shedding light on the relationship between UV damage and the nucleoplasmic response. For instance, it would be of particular interest to further investigate how disruption of the DDB2–PCNA interaction affects transcriptional regulation and the DNA damage response more broadly. Future studies integrating high-resolution structural analysis of γ-H2AX foci with sequencing-based technologies to assess chromatin composition and conformation (Natale et al. 2017; Schuette et al. 2025; Gjoni et al. 2025), nucleosomes density (Abdulhay et al. 2023; Wen et al. 2025; Chen et al. 2025), and chromatin loops (Liefsoens et al. 2024; Kim et al. 2025) may contribute to elucidating the precise molecular mechanisms by which these processes promote pathogenesis and may help identify potential therapeutic targets.

## Supplementary Information

Below is the link to the electronic supplementary material.

Supplementary file1 (PDF 378 KB)

## Data Availability

The datasets used and analyzed during the present study are available from the corresponding author upon reasonable request.

## References

[CR1] Abdulhay NJ, Hsieh LJ, McNally CP et al (2023) Nucleosome density shapes kilobase-scale regulation by a mammalian chromatin remodeler. Nat Struct Mol Biol 30:1571–1581. 10.1038/s41594-023-01093-6 37696956 10.1038/s41594-023-01093-6 PMC10584690

[CR2] Altıntaş UB, Seo J-H, Giambartolomei C et al (2024) Decoding the epigenetics and chromatin loop dynamics of androgen receptor-mediated transcription. Nat Commun 15:9494. 10.1038/s41467-024-53758-5 39489778 10.1038/s41467-024-53758-5 PMC11532539

[CR3] Ambrosio S, Noviello A, Di Fusco G et al (2025) Interplay and dynamics of chromatin architecture and DNA damage response: an overview. Cancers (Basel) 17:949. 10.3390/cancers17060949 40149285 10.3390/cancers17060949 PMC11940107

[CR4] Aricthota S, Rana PP, Haldar D (2022) Histone acetylation dynamics in repair of DNA double-strand breaks. Front Genet 13:926577. 10.3389/fgene.2022.926577 36159966 10.3389/fgene.2022.926577 PMC9503837

[CR5] Biggiogera M, Cavallo M, Casali C (2024) A brief history of the Feulgen reaction. Histochem Cell Biol. 10.1007/s00418-024-02279-9 38609528 10.1007/s00418-024-02279-9 PMC11227455

[CR6] Burgess RC, Misteli T, Oberdoerffer P (2012) DNA damage, chromatin, and transcription: the trinity of aging. Curr Opin Cell Biol 24:724–730. 10.1016/j.ceb.2012.07.005 22902297 10.1016/j.ceb.2012.07.005 PMC3524355

[CR7] Casali C, Siciliani S, Galgano L, Biggiogera M (2022a) Oxidative stress and nuclear reprogramming: a pilot study of the effects of reactive oxygen species on architectural and epigenetic landscapes. Int J Mol Sci 24:153. 10.3390/ijms24010153 36613595 10.3390/ijms24010153 PMC9820425

[CR8] Casali C, Siciliani S, Zannino L, Biggiogera M (2022b) Histochemistry for nucleic acid research: 60 years in the European Journal of Histochemistry. Eur J Histochem 66:3409. 10.4081/ejh.2022.3409 35441834 10.4081/ejh.2022.3409 PMC9044459

[CR9] Casali C, Galgano L, Zannino L et al (2024) Impact of heat and cold shock on epigenetics and chromatin structure. Eur J Cell Biol 103:151373. 10.1016/j.ejcb.2023.151373 38016352 10.1016/j.ejcb.2023.151373

[CR10] Casali C, Cavallo M, Diaf A et al (2026) Now you see me: visualizing nuclear complexity by selective staining. Methods 250:46–54. 10.1016/j.ymeth.2026.03.005 41831750 10.1016/j.ymeth.2026.03.005

[CR11] Cavallo M, Diaf A, Milanesi G et al (2026) Resolving sub-nuclear architecture from compartments to functional domains. Int J Mol Sci 27:4680. 10.3390/ijms27114680 42278215 10.3390/ijms27114680 PMC13256304

[CR12] Cazzalini O, Perucca P, Mocchi R et al (2014) DDB2 association with PCNA is required for its degradation after UV-induced DNA damage. Cell Cycle 13:240–248. 10.4161/cc.26987 24200966 10.4161/cc.26987 PMC3906241

[CR13] Chen L, Maristany MJ, Farr SE et al (2025) Nucleosome spacing can fine-tune higher-order chromatin assembly. Nat Commun 16:6315. 10.1038/s41467-025-61482-x 40628730 10.1038/s41467-025-61482-x PMC12238351

[CR14] Contreras L, García-Gaipo L, Casar B, Gandarillas A (2024) DNA damage signalling histone H2AX is required for tumour growth. Cell Death Discov 10:99. 10.1038/s41420-024-01869-9 38402225 10.1038/s41420-024-01869-9 PMC10894207

[CR15] Cowell IG, Sunter NJ, Singh PB et al (2007) γH2AX foci form preferentially in euchromatin after ionising-radiation. PLoS ONE 2:e1057. 10.1371/journal.pone.0001057 17957241 10.1371/journal.pone.0001057 PMC2020439

[CR16] Dardare J, Witz A, Betz M et al (2024) DDB2 expression lights the way for precision radiotherapy response in PDAC cells, with or without olaparib. Cell Death Discov 10:411. 10.1038/s41420-024-02188-9 39333096 10.1038/s41420-024-02188-9 PMC11436999

[CR17] Davidovich A, Cuomo D, Su H et al (2026) Non-Mendelian inheritance of DNA methylation patterns in mice. Nat Genet. 10.1038/s41588-026-02604-z 42162411 10.1038/s41588-026-02604-z PMC13263155

[CR18] Dibitetto D, Liptay M, Vivalda F et al (2024) H2AX promotes replication fork degradation and chemosensitivity in BRCA-deficient tumours. Nat Commun 15:4430. 10.1038/s41467-024-48715-1 38789420 10.1038/s41467-024-48715-1 PMC11126719

[CR19] Donnio L-M, Giglia-Mari G (2025) Keep calm and reboot – How cells restart transcription after DNA damage and DNA repair. FEBS Lett 599:275–294. 10.1002/1873-3468.14964 38991979 10.1002/1873-3468.14964 PMC11771587

[CR20] Downs JA, Gasser SM (2024) Chromatin remodeling and spatial concerns in DNA double-strand break repair. Curr Opin Cell Biol 90:102405. 10.1016/j.ceb.2024.102405 39083951 10.1016/j.ceb.2024.102405

[CR21] Fakan S, van Driel R (2007) The perichromatin region: a functional compartment in the nucleus that determines large-scale chromatin folding. Semin Cell Dev Biol 18:676–681. 10.1016/j.semcdb.2007.08.010 17920313 10.1016/j.semcdb.2007.08.010

[CR22] Fedkenheuer M, Shang Y, Jung S et al (2025) A dual role of cohesin in DNA DSB repair. Nat Commun 16:843. 10.1038/s41467-025-56086-4 39833168 10.1038/s41467-025-56086-4 PMC11747280

[CR23] Fernandez-De-Los-Reyes I, Gomez-Dorronsoro M, Monreal-Santesteban I et al (2023) ZEB1 hypermethylation is associated with better prognosis in patients with colon cancer. Clin Epigenetics 15:193. 10.1186/s13148-023-01605-7 38093305 10.1186/s13148-023-01605-7 PMC10720242

[CR24] Furuya K, Ikura M, Ikura T (2023) Machine learning extracts oncogenic-specific γ-H2AX foci formation pattern upon genotoxic stress. Genes Cells 28:237–243. 10.1111/gtc.13005 36565298 10.1111/gtc.13005

[CR25] Gershman A, Sauria MEG, Guitart X et al (2022) Epigenetic patterns in a complete human genome. Science 376:eabj5089. 10.1126/science.abj5089 35357915 10.1126/science.abj5089 PMC9170183

[CR26] Gjoni K, Gunsalus LM, Kuang S et al (2025) Comparing chromatin contact maps at scale: methods and insights. Nat Methods 22:824–833. 10.1038/s41592-025-02630-5 40108448 10.1038/s41592-025-02630-5 PMC11978506

[CR27] Gorbunova V, Seluanov A, Mao Z, Hine C (2007) Changes in DNA repair during aging. Nucleic Acids Res 35:7466–7474. 10.1093/nar/gkm756 17913742 10.1093/nar/gkm756 PMC2190694

[CR28] Halsall JA, Andrews S, Krueger F et al (2021) Histone modifications form a cell-type-specific chromosomal bar code that persists through the cell cycle. Sci Rep 11:3009. 10.1038/s41598-021-82539-z 33542322 10.1038/s41598-021-82539-z PMC7862352

[CR29] Hustedt N, Durocher D (2017) The control of DNA repair by the cell cycle. Nat Cell Biol 19:1–9. 10.1038/ncb3452 10.1038/ncb3452 28008184

[CR30] Jalan M, Brambati A, Shah H et al (2025) RNA transcripts serve as a template for double-strand break repair in human cells. Nat Commun 16:4349. 10.1038/s41467-025-59510-x 40348775 10.1038/s41467-025-59510-x PMC12065846

[CR31] Jeon Y, Lu Y, Ferrari MM et al (2024) RNA-mediated double-strand break repair by end-joining mechanisms. Nat Commun 15:7935. 10.1038/s41467-024-51457-9 39261460 10.1038/s41467-024-51457-9 PMC11390984

[CR32] Kaya VO, Adebali O (2025) UV-induced reorganization of 3D genome mediates DNA damage response. Nat Commun 16:1376. 10.1038/s41467-024-55724-7 39910043 10.1038/s41467-024-55724-7 PMC11799157

[CR33] Kelliher JL, Folkerts ML, Shen KV et al (2024) Evolved histone tail regulates 53BP1 recruitment at damaged chromatin. Nat Commun 15:4634. 10.1038/s41467-024-49071-w 38821984 10.1038/s41467-024-49071-w PMC11143218

[CR34] Kikuchi M, Morita S, Wakamori M et al (2023) Epigenetic mechanisms to propagate histone acetylation by p300/CBP. Nat Commun 14:4103. 10.1038/s41467-023-39735-4 37460559 10.1038/s41467-023-39735-4 PMC10352329

[CR35] Kim IV, Navarrete C, Grau-Bové X et al (2025) Chromatin loops are an ancestral hallmark of the animal regulatory genome. Nature 642:1097–1105. 10.1038/s41586-025-08960-w 40335694 10.1038/s41586-025-08960-w PMC12221973

[CR36] Kreibich E, Krebs AR (2023) Relevance of DNA methylation at enhancers for the acquisition of cell identities. FEBS Lett 597:1805–1817. 10.1002/1873-3468.14686 37343149 10.1002/1873-3468.14686

[CR37] Liefsoens M, Földes T, Barbi M (2024) Spectral-based detection of chromatin loops in multiplexed super-resolution FISH data. Nat Commun 15:7670. 10.1038/s41467-024-51650-w 39237524 10.1038/s41467-024-51650-w PMC11377450

[CR38] Lin M-Y, Lo Y-C, Hung J-H (2025) Unveiling chromatin dynamics with virtual epigenome. Nat Commun 16:3491. 10.1038/s41467-025-58481-3 40221401 10.1038/s41467-025-58481-3 PMC11993739

[CR39] Lindner P, Paul S, Eckstein M et al (2020) EMT transcription factor ZEB1 alters the epigenetic landscape of colorectal cancer cells. Cell Death Dis 11:147. 10.1038/s41419-020-2340-4 32094334 10.1038/s41419-020-2340-4 PMC7040187

[CR40] Liu Y, Li H, Wilson CN et al (2022) Histone H2AX promotes metastatic progression by preserving glycolysis via hexokinase-2. Sci Rep 12:3758. 10.1038/s41598-022-07675-6 35260660 10.1038/s41598-022-07675-6 PMC8904825

[CR41] Liu X, Guo C, Leng T et al (2023) Differential regulation of H3K9/H3K14 acetylation by small molecules drives neuron-fate-induction of glioma cell. Cell Death Dis 14:142. 10.1038/s41419-023-05611-8 36805688 10.1038/s41419-023-05611-8 PMC9941105

[CR42] López-Hernández L, Toolan-Kerr P, Bannister AJ, Millán-Zambrano G (2025) Dynamic histone modification patterns coordinating DNA processes. Mol Cell 85:225–237. 10.1016/j.molcel.2024.10.034 39824165 10.1016/j.molcel.2024.10.034

[CR43] Lorat Y, Schanz S, Schuler N et al (2012) Beyond repair foci: DNA double-strand break repair in euchromatic and heterochromatic compartments analyzed by transmission electron microscopy. PLoS ONE 7:e38165. 10.1371/journal.pone.0038165 22666473 10.1371/journal.pone.0038165 PMC3364237

[CR44] Mathis N, Allam A, Tálas A et al (2025) Machine learning prediction of prime editing efficiency across diverse chromatin contexts. Nat Biotechnol 43:712–719. 10.1038/s41587-024-02268-2 38907037 10.1038/s41587-024-02268-2 PMC7617539

[CR45] Mattei AL, Bailly N, Meissner A (2022) DNA methylation: a historical perspective. Trends Genet 38:676–707. 10.1016/j.tig.2022.03.010 35504755 10.1016/j.tig.2022.03.010

[CR46] Millán-Zambrano G, Burton A, Bannister AJ, Schneider R (2022) Histone post-translational modifications—Cause and consequence of genome function. Nat Rev Genet 23:563–580. 10.1038/s41576-022-00468-7 35338361 10.1038/s41576-022-00468-7

[CR47] Min S, Ji J-H, Heo Y, Cho H (2022) Transcriptional regulation and chromatin dynamics at DNA double-strand breaks. Exp Mol Med 54:1705–1712. 10.1038/s12276-022-00862-5 36229590 10.1038/s12276-022-00862-5 PMC9636152

[CR48] Natale F, Rapp A, Yu W et al (2017) Identification of the elementary structural units of the DNA damage response. Nat Commun 8:15760. 10.1038/ncomms15760 28604675 10.1038/ncomms15760 PMC5472794

[CR49] Oizumi T, Suzuki T, Kobayashi J, Nakamura AJ (2024) Senescence-associated heterochromatin foci suppress γ-H2AX focus formation induced by radiation exposure. Int J Mol Sci 25:3355. 10.3390/ijms25063355 38542327 10.3390/ijms25063355 PMC10969922

[CR50] Ortega P, Gómez-González B, Aguilera A (2021) Heterogeneity of DNA damage incidence and repair in different chromatin contexts. DNA Repair 107:103210. 10.1016/j.dnarep.2021.103210 34416542 10.1016/j.dnarep.2021.103210

[CR51] Padeken J, Methot SP, Gasser SM (2022) Establishment of H3K9-methylated heterochromatin and its functions in tissue differentiation and maintenance. Nat Rev Mol Cell Biol 23:623–640. 10.1038/s41580-022-00483-w 35562425 10.1038/s41580-022-00483-w PMC9099300

[CR52] Park J, Lee K, Kim K, Yi S-J (2022) The role of histone modifications: from neurodevelopment to neurodiseases. Sig Transduct Target Ther 7:217. 10.1038/s41392-022-01078-9 10.1038/s41392-022-01078-9 PMC9259618 35794091

[CR53] Paull TT, Opresko PL (2026) Epigenetic consequences of DNA damage. Mol Cell 86:439–448. 10.1016/j.molcel.2025.12.029 41544625 10.1016/j.molcel.2025.12.029 PMC12820771

[CR54] Perucca P, Sommatis S, Mocchi R et al (2015) A DDB2 mutant protein unable to interact with PCNA promotes cell cycle progression of human transformed embryonic kidney cells. Cell Cycle 14:3920–3928. 10.1080/15384101.2015.1120921 26697842 10.1080/15384101.2015.1120921 PMC4825770

[CR55] Perucca P, Mocchi R, Guardamagna I et al (2018) A damaged DNA binding protein 2 mutation disrupting interaction with proliferating-cell nuclear antigen affects DNA repair and confers proliferation advantage. Biochim Biophys Acta Mol Cell Res 1865:898–907. 10.1016/j.bbamcr.2018.03.012 29604309 10.1016/j.bbamcr.2018.03.012

[CR56] Perucca P, Bassi E, Vetro M et al (2024) Epithelial-to-mesenchymal transition and NF-kB pathways are promoted by a mutant form of DDB2, unable to bind PCNA, in UV-damaged human cells. BMC Cancer 24:616. 10.1186/s12885-024-12368-6 38773406 10.1186/s12885-024-12368-6 PMC11110260

[CR57] Plessier A, Chansard A, Petit E et al (2026) Proteomic profiling of UV damage repair patches uncovers histone chaperones with central functions in chromatin repair. Nat Commun 17:2127. 10.1038/s41467-026-68781-x 41605964 10.1038/s41467-026-68781-x PMC12957454

[CR58] Policarpi C, Munafò M, Tsagkris S et al (2024) Systematic epigenome editing captures the context-dependent instructive function of chromatin modifications. Nat Genet 56:1168–1180. 10.1038/s41588-024-01706-w 38724747 10.1038/s41588-024-01706-w PMC11176084

[CR59] Polyzos AA, Cheong A, Yoo JH et al (2024) Base excision repair and double strand break repair cooperate to modulate the formation of unrepaired double strand breaks in mouse brain. Nat Commun 15:7726. 10.1038/s41467-024-51906-5 39231940 10.1038/s41467-024-51906-5 PMC11375129

[CR60] Prabhu KS, Kuttikrishnan S, Ahmad N et al (2024) H2AX: a key player in DNA damage response and a promising target for cancer therapy. Biomed Pharmacother 175:116663. 10.1016/j.biopha.2024.116663 38688170 10.1016/j.biopha.2024.116663

[CR61] Rawal CC, Butova NL, Mitra A, Chiolo I (2022) An expanding toolkit for heterochromatin repair studies. Genes 13:529. 10.3390/genes13030529 35328082 10.3390/genes13030529 PMC8955653

[CR62] Rueden CT, Schindelin J, Hiner MC et al (2017) ImageJ2: ImageJ for the next generation of scientific image data. BMC Bioinformatics 18:529. 10.1186/s12859-017-1934-z 29187165 10.1186/s12859-017-1934-z PMC5708080

[CR63] Saxena S, Zou L (2022) Hallmarks of DNA replication stress. Mol Cell 82:2298–2314. 10.1016/j.molcel.2022.05.004 35714587 10.1016/j.molcel.2022.05.004 PMC9219557

[CR64] Schuette G, Lao Z, Zhang B (2025) Chromogen: diffusion model predicts single-cell chromatin conformations. Sci Adv 11:eadr8265. 10.1126/sciadv.adr8265 39888999 10.1126/sciadv.adr8265 PMC11784829

[CR65] Silva E, Ideker T (2019) Transcriptional responses to DNA damage. DNA Repair (Amst) 79:40–49. 10.1016/j.dnarep.2019.05.002 31102970 10.1016/j.dnarep.2019.05.002 PMC6570417

[CR66] Steurer B, Janssens RC, Geijer ME et al (2022) DNA damage-induced transcription stress triggers the genome-wide degradation of promoter-bound Pol II. Nat Commun 13:3624. 10.1038/s41467-022-31329-w 35750669 10.1038/s41467-022-31329-w PMC9232492

[CR67] Strzałka W, Zgłobicki P, Kowalska E et al (2020) The dark side of UV-induced DNA lesion repair. Genes (Basel) 11:1450. 10.3390/genes11121450 33276692 10.3390/genes11121450 PMC7761550

[CR68] Valceski M, Engels E, Vogel S et al (2024) A novel approach to double-strand DNA break analysis through γ-H2AX confocal image quantification and bio-dosimetry. Sci Rep 14:27591. 10.1038/s41598-024-76683-5 39528587 10.1038/s41598-024-76683-5 PMC11554680

[CR69] Valdiglesias V, Giunta S, Fenech M et al (2013) γH2AX as a marker of DNA double strand breaks and genomic instability in human population studies. Mutat Res 753:24–40. 10.1016/j.mrrev.2013.02.001 23416207 10.1016/j.mrrev.2013.02.001

[CR70] Vergara X, Manjón AG, de Haas M et al (2024) Widespread chromatin context-dependencies of DNA double-strand break repair proteins. Nat Commun 15:5334. 10.1038/s41467-024-49232-x 38909016 10.1038/s41467-024-49232-x PMC11193718

[CR71] Wang Y, Tsukioka D, Oda S et al (2024) Involvement of H2A variants in DNA damage response of zygotes. Cell Death Discov 10:1–10. 10.1038/s41420-024-01999-0 38744857 10.1038/s41420-024-01999-0 PMC11094039

[CR72] Watt K, Dauber B, Szkop KJ et al (2025) Epigenetic alterations facilitate transcriptional and translational programs in hypoxia. Nat Cell Biol 27:1965–1981. 10.1038/s41556-025-01786-8 41102449 10.1038/s41556-025-01786-8 PMC12611764

[CR73] Wen Z, Fang R, Zhang R et al (2025) Nucleosome wrapping states encode principles of 3D genome organization. Nat Commun 16:352. 10.1038/s41467-024-54735-8 39753536 10.1038/s41467-024-54735-8 PMC11699143

[CR74] Wensveen MR, Dixit AA, van Schendel R et al (2024) Double-strand breaks in facultative heterochromatin require specific movements and chromatin changes for efficient repair. Nat Commun 15:8984. 10.1038/s41467-024-53313-2 39419979 10.1038/s41467-024-53313-2 PMC11487122

[CR75] Weyemi U, Redon CE, Bonner WM (2016a) H2AX and EMT: deciphering beyond DNA repair. Cell Cycle 15:1305–1306. 10.1080/15384101.2016.1160659 26986807 10.1080/15384101.2016.1160659 PMC4889283

[CR76] Weyemi U, Redon CE, Choudhuri R et al (2016b) The histone variant H2A.X is a regulator of the epithelial-mesenchymal transition. Nat Commun 7:10711. 10.1038/ncomms10711 26876487 10.1038/ncomms10711 PMC4756313

[CR77] Wu X, Zhang X, Tang S, Wang Y (2025) The important role of the histone acetyltransferases p300/CBP in cancer and the promising anticancer effects of p300/CBP inhibitors. Cell Biol Toxicol 41:32. 10.1007/s10565-024-09984-0 39825161 10.1007/s10565-024-09984-0 PMC11742294

[CR78] Yang JH, Brandão HB, Hansen AS (2023) DNA double-strand break end synapsis by DNA loop extrusion. Nat Commun 14:1913. 10.1038/s41467-023-37583-w 37024496 10.1038/s41467-023-37583-w PMC10079674

[CR79] Yousefzadeh M, Henpita C, Vyas R et al (2021) DNA damage—How and why we age? Elife 10:e62852. 10.7554/eLife.62852 33512317 10.7554/eLife.62852 PMC7846274

[CR80] Zannino L, Casali C, Siciliani S, Biggiogera M (2021) The dynamics of the nuclear environment and their impact on gene function. J Biochem 169:259–264. 10.1093/jb/mvaa091 32745171 10.1093/jb/mvaa091

[CR81] Zannino L, Pagano A, Casali C et al (2023) Mercury chloride alters heterochromatin domain organization and nucleolar activity in mouse liver. Histochem Cell Biol 159:61–76. 10.1007/s00418-022-02151-8 36136163 10.1007/s00418-022-02151-8 PMC9899742

[CR82] Zencir S, Dilg D, Bruzzone MJ et al (2025) A two-step regulatory mechanism dynamically controls histone H3 acetylation by SAGA complex at growth-related promoters. Nucleic Acids Res 53:gkaf276. 10.1093/nar/gkaf276 40207626 10.1093/nar/gkaf276 PMC11983098

[CR83] Zhang J, Fan X, Zhou Y et al (2022) The PRMT5-LSD1 axis confers Slug dual transcriptional activities and promotes breast cancer progression. J Exp Clin Cancer Res 41:191. 10.1186/s13046-022-02400-7 35655230 10.1186/s13046-022-02400-7 PMC9164399

[CR84] Zhao S, Lu J, Pan B et al (2023) TNRC18 engages H3K9me3 to mediate silencing of endogenous retrotransposons. Nature 623:633–642. 10.1038/s41586-023-06688-z 37938770 10.1038/s41586-023-06688-z PMC11000523

[CR85] Zhou L, Chen Z, Zou Y et al (2025) ASB7 is a negative regulator of H3K9me3 homeostasis. Science. 10.1126/science.adq7408 40440427 10.1126/science.adq7408

