# Supplementary material for: Epigenetic remodeling during UV exposure: high resolution analysis of histone post-translational modifications in a DNA binding protein 2 mutant model
Source: Histochem Cell Biol. 2026 Jul 15;164(1):60. doi: 10.1007/s00418-026-02514-5 (PMC13372869; doi:10.1007/s00418-026-02514-5)

Epigenetic Remodeling During UV Exposure: High Resolution Analysis of Histone Post-Translational Modifications in a DNA Binding Protein 2 Mutant Model

Claudio Casali[1*], Margherita Cavallo[1], Adel Diaf[1], Davide Tunesi[1], Martina Furfaro[2], Anna Tricarico[2], Gloria Milanesi[1], Paola Perucca[2], Ornella Cazzalini[2], Marco Biggiogera[1]

1. Department of Biology and Biotechnology, Laboratory of Cell Biology and Neurobiology, University of Pavia, Pavia, Italy.
2. Department of Molecular Medicine, Immunology and General Pathology Unit, University of Pavia, Pavia, Italy.

Ornella Cazzalini and Marco Biggiogera contributed equally to this work.
Correspondence: Claudio Casali.
claudio.casali@unipv.it

Supplementary Data 1.

Exact *P* values.

Fig. 1 CTR 3d vs $DDB2^{Wt}$ 3d: 0.0523; $DDB2^{Wt}$ 3d vs $DDB2^{PCNA-}$ 3d: <0.0001; CTR 3d vs $DDB2^{PCNA-}$ 3d: 0.0024; CTR 7d vs $DDB2^{Wt}$ 7d: 0.0315; $DDB2^{Wt}$ 7d vs $DDB2^{PCNA-}$ 7d: 0.0004; CTR 7d vs $DDB2^{PCNA-}$ 7d: 0.3216.

Fig. 2 CTR 3d vs $DDB2^{Wt}$ 3d: 0.8526; $DDB2^{Wt}$ 3d vs $DDB2^{PCNA-}$ 3d: <0.0001; CTR 3d vs $DDB2^{PCNA-}$ 3d: 0.0002; CTR 7d vs $DDB2^{Wt}$ 7d: 0.9495; $DDB2^{Wt}$ 7d vs $DDB2^{PCNA-}$ 7d: <0.0001; CTR 7d vs $DDB2^{PCNA-}$ 7d: <0.0001.

Fig. 3 (**b**) CTR No UV vs $DDB2^{Wt}$ No UV: >0.9999; CTR No UV vs $DDB2^{PCNA-}$ No UV: >0.9999; $DDB2^{Wt}$ No UV vs $DDB2^{PCNA-}$ No UV: >0.9999; CTR 3d vs $DDB2^{Wt}$ 3d: 0.0465; CTR 3d vs $DDB2^{PCNA-}$ 3d: <0.0001; $DDB2^{Wt}$ 3d vs $DDB2^{PCNA-}$ 3d: 0.0051; CTR 7d vs $DDB2^{Wt}$ 7d: 0.5511; CTR 7d vs $DDB2^{PCNA-}$ 7d: <0.0001; $DDB2^{Wt}$ 7d vs $DDB2^{PCNA-}$ 7d: <0.0001.

Fig. 3 (**c**) CTR 3d vs $DDB2^{Wt}$ 3d: 0.0001; $DDB2^{Wt}$ 3d vs $DDB2^{PCNA-}$ 3d: <0.0006; CTR 3d vs $DDB2^{PCNA-}$ 3d: <0.0001; CTR 7d vs $DDB2^{Wt}$ 7d: 0.9004; $DDB2^{Wt}$ 7d vs $DDB2^{PCNA-}$ 7d: <0.0001; CTR 7d vs $DDB2^{PCNA-}$ 7d: <0.0001.

Fig. 4 CTR 3d vs $DDB2^{Wt}$ 3d: 0.0002; $DDB2^{Wt}$ 3d vs $DDB2^{PCNA-}$ 3d: <0.0001; CTR 3d vs $DDB2^{PCNA-}$ 3d: <0.0001; CTR 7d vs $DDB2^{Wt}$ 7d: 0.0653; $DDB2^{Wt}$ 7d vs $DDB2^{PCNA-}$ 7d: 0.0989; CTR 7d vs $DDB2^{PCNA-}$ 7d: 0.0003.

Fig. 5 CTR No UV vs CTR 3d: <0.0001; $DDB2^{Wt}$ No UV vs $DDB2^{Wt}$ 3d: <0.0001; CTR 3d vs $DDB2^{PCNA-}$ 3d: 0.089. CTR No UV vs $DDB2^{Wt}$ No UV: >0.9999; CTR No UV vs $DDB2^{PCNA-}$ No UV: >0.9999; $DDB2^{Wt}$ No UV vs $DDB2^{PCNA-}$ No UV: >0.9999; CTR 1h UV vs $DDB2^{Wt}$ 1h UV: >0.9999; CTR 1h UV vs $DDB2^{PCNA-}$ 1h UV: >0.9999; $DDB2^{Wt}$ 1h UV vs $DDB2^{PCNA-}$ 1h UV: >0.9999; CTR 3d UV vs $DDB2^{Wt}$ 3d UV: 0.8239; $DDB2^{Wt}$ 3d UV vs $DDB2^{PCNA-}$ 3d UV: 0.3905.

Fig. 6 (**b**) CTR 1h vs $DDB2^{Wt}$ 1h: 0.7142; $DDB2^{Wt}$ 1h vs $DDB2^{PCNA-}$ 1h: 0.9917; CTR 1h vs $DDB2^{PCNA-}$ 1h: 0.7864; CTR 72h vs $DDB2^{Wt}$ 72h: 0.1183; $DDB2^{Wt}$ 72h vs $DDB2^{PCNA-}$ 72h: 0.2443; CTR 72h vs $DDB2^{PCNA-}$ 72h: 0.0025.

Fig. 6 (**d**) CTR No UV vs $DDB2^{Wt}$ No UV: 0.5327; $DDB2^{Wt}$ No UV vs $DDB2^{PCNA-}$ No UV: 0.7621; CTR No UV vs $DDB2^{PCNA-}$ No UV: 0.9452; CTR 3d vs $DDB2^{Wt}$ 3d: 0.0179; $DDB2^{Wt}$ 3d vs $DDB2^{PCNA-}$ 3d: 0.9873; CTR 3d vs $DDB2^{PCNA-}$ 3d: 0.0345; CTR 7d vs $DDB2^{Wt}$ 7d: 0.7271; $DDB2^{Wt}$ 7d vs $DDB2^{PCNA-}$ 7d: 0.6832; CTR 7d vs $DDB2^{PCNA-}$ 7d: 0.3143.

Fig. 6 (**e**) CTR: No UV vs 3d: 0.6654; 3d vs 7d: 0.7301; No UV vs 7d: 0.3569; $DDB2^{Wt}$: No UV vs 3d: 0.0768; 3d vs 7d: 0.1922; No UV vs 7d: 0.8480; $DDB2^{PCNA-}$: No UV vs 3d: 0.0212; 3d vs 7d: 0.2430; No UV vs 7d: 0.2408.

Fig. 6 (**f**) Euchromatin + PR vs Heterochromatin in CTR: 0.4096; Euchromatin + PR vs Heterochromatin in $DDB2^{Wt}$: 0.3261; Euchromatin + PR vs Heterochromatin in $DDB2^{PCNA-}$: 0.0326.

Fig. 7 (**a**) CTR: No UV vs 3d: 0.7690; 3d vs 7d: 0.9837; No UV vs 7d: 0.8624; $DDB2^{Wt}$: No UV vs 3d: 0.0523; 3d vs 7d: >0.9999; No UV vs 7d: 0.0519; $DDB2^{PCNA-}$: No UV vs 3d: <0.0001; 3d vs 7d: 0.0079; No UV vs 7d: 0.0381.

Fig. 7 (**b**) CTR: No UV vs 3d: 0.0004; 3d vs 7d: 0.9715; No UV vs 7d: 0.0007; DDB2^{Wt}: No UV vs 3d: <0.0001; 3d vs 7d: 0.5821; No UV vs 7d: <0.0001; DDB2^{PCNA-}: No UV vs 3d: 0.9967; 3d vs 7d: 0.0343; No UV vs 7d: 0.0408.

Fig. 7 (**c**) CTR: No UV vs 3d: 0.7026; 3d vs 7d: 0.9495; No UV vs 7d: 0.8724; DDB2^{Wt}: No UV vs 3d: <0.0001; 3d vs 7d: <0.0001; No UV vs 7d: 0.7156; DDB2^{PCNA-}: No UV vs 3d: <0.0001; 3d vs 7d: 0.0693; No UV vs 7d: <0.0001.

Fig. 7 (**d**) CTR: No UV vs 3d: 0.0016; 3d vs 7d: 0.7145; No UV vs 7d: 0.0002; DDB2^{Wt}: No UV vs 3d: 0.6760; 3d vs 7d: 0.6939; No UV vs 7d: 0.9995; DDB2^{PCNA-}: No UV vs 3d: 0.0021; 3d vs 7d: 0.2227; No UV vs 7d: 0.1101.

Fig. 7 (**e**) CTR: No UV vs 1h: 0.1240; 1h vs 72h: 0.0133; No UV vs 72h: <0.0001; DDB2^{Wt}: No UV vs 1h: 0.1220; 1h vs 72h: 0.9502; No UV vs 72h: 0.2119; DDB2^{PCNA-}: No UV vs 1h: 0.0448; 1h vs 72h: 0.0218; No UV vs 72h: <0.0001.

Supplementary Figure 1 CTR No UV vs DDB2^{Wt} No UV: >0.9999; CTR No UV vs DDB2^{PCNA-} No UV: >0.9999; DDB2^{Wt} No UV vs DDB2^{PCNA-} No UV: >0.9999; CTR 1d UV vs DDB2^{Wt} 1d UV: >0.9999; CTR 1d UV vs DDB2^{PCNA-} 1d UV: >0.9999; DDB2^{Wt} 1d UV vs DDB2^{PCNA-} 1d UV: 0.4509; CTR 2d UV vs DDB2^{Wt} 2d UV: 0.9809; CTR 2d UV vs DDB2^{PCNA-} 2d UV: 0.0938; DDB2^{Wt} 2d UV vs DDB2^{PCNA-} 2d UV: 0.1355; CTR 3d UV vs DDB2^{Wt} 3d UV: 0.9947; CTR 3d UV vs DDB2^{PCNA-} 3d UV: 0.1100; DDB2^{Wt} 3d UV vs DDB2^{PCNA-} 3d UV: 0.8228; CTR 4d UV vs DDB2^{Wt} 4d UV: >0.9999; CTR 4d UV vs DDB2^{PCNA-} 4d UV: 0.2336; DDB2^{Wt} 4d UV vs DDB2^{PCNA-} 4d UV: 0.3308.

Supplementary Figure 1.

Cell growth analysis performed in HEK293 DDB2^{Wt} or DDB2^{PCNA-}. (**a**) Daily cell counting up to 7 days after UV damage induction P values: CTR 7d vs DDB2^{PCNA-} 7d: <0.0001; DDB2^{Wt} 7d vs DDB2^{PCNA-} 7d: < 0.0001. (**b**) Cytofluorimetric analysis (x-axis: DNA; y-axis: cell count) of S phase before and after 3d irradiation. S phase: HEK293 DDB2^{Wt} = 26%; HEK293 DDB2^{Wt} 3d UV = 24%; HEK293 DDB2^{PCNA-} = 29%; HEK293 DDB2^{PCNA-} UV = 22%.

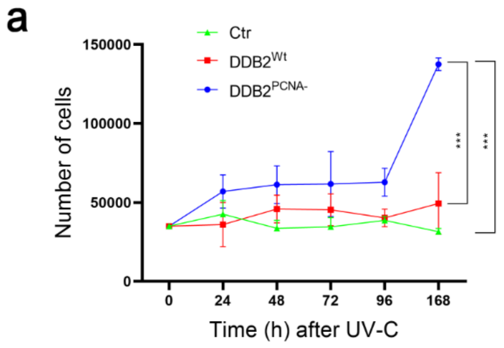

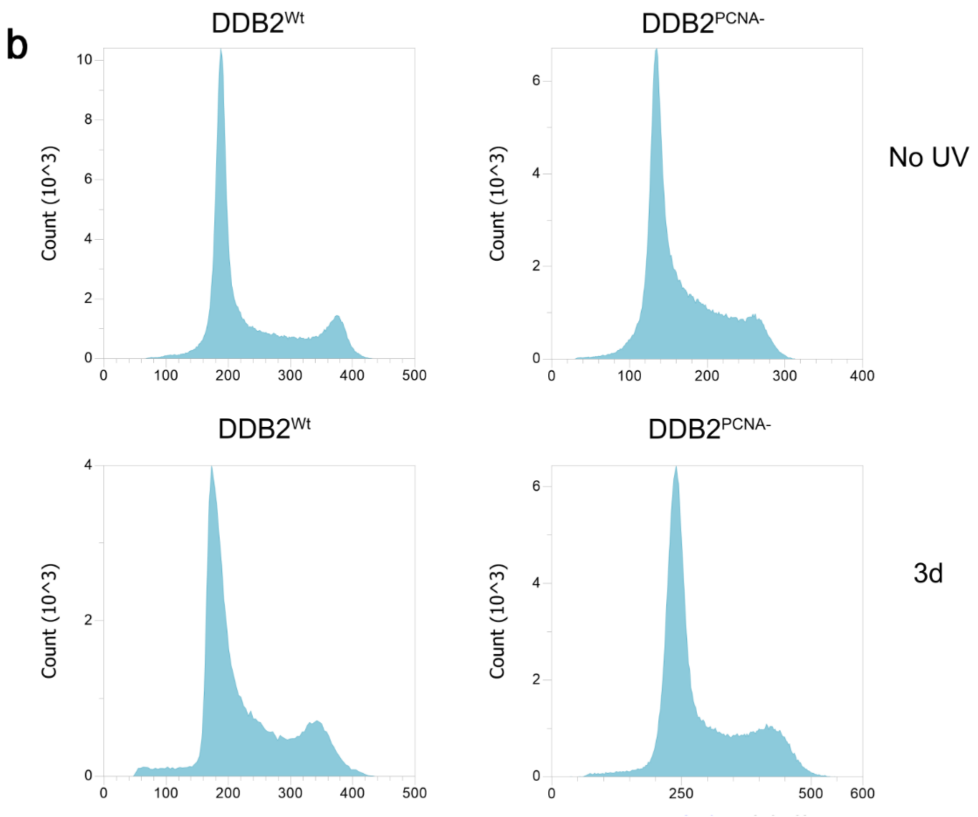

Supplementary Figure 2.

Representative Western blot data to validate DDB2 expression level. (**a**) Ponceau S staining. (**b**) DDB2 and Actin, used as housekeeping protein. Molecular weights are intended in KDa.

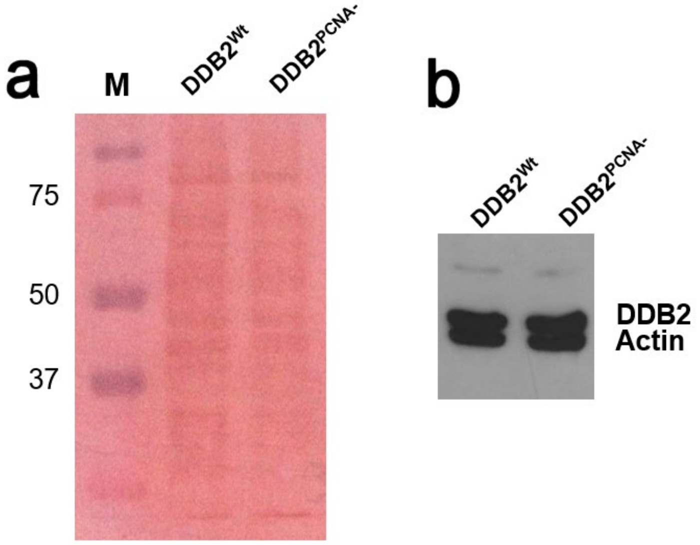

Supplementary Figure 3.

Representative Western blot data for the quantifications of H3K9me3 abundance shown in Figure 3. (**a**) Ponceau S staining. (**b**) Actin used as housekeeping protein. (**c**) H3K9me3 (arrow). Molecular weights are intended in KDa.

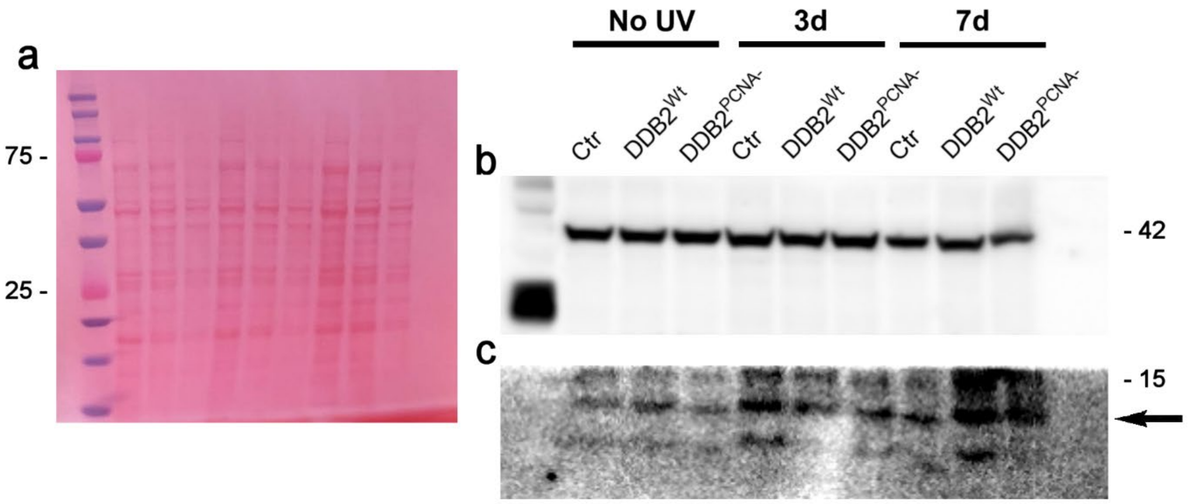

Supplement: Supplementary file 1 — Supplementary file1 (PDF 378 KB) [file 418_2026_2514_MOESM1_ESM.pdf]
